# Two avian *Plasmodium* species trigger different transcriptional responses on their vector *Culex pipiens*

**DOI:** 10.1111/mec.17240

**Published:** 2023-12-18

**Authors:** Marta Garrigós, Guillem Ylla, Josué Martínez‐de la Puente, Jordi Figuerola, María José Ruiz‐López

**Affiliations:** ^1^ Department of Parasitology University of Granada Granada Spain; ^2^ Bioinformatics and Genome Biology Lab, Faculty of Biochemistry, Biophysics and Biotechnology Jagiellonian University Krakow Poland; ^3^ CIBER Epidemiologia y Salud Pública (CIBERESP) Madrid Spain; ^4^ Department of Wetland Ecology Estación Biológica de Doñana, CSIC Sevilla Spain

**Keywords:** avian malaria, mosquito transcriptome, *Plasmodium cathemerium*, *Plasmodium relictum*, RNAseq, vector‐borne parasites

## Abstract

Malaria is a mosquito‐borne disease caused by protozoans of the genus *Plasmodium* that affects both humans and wildlife. The fitness consequences of infections by avian malaria are well known in birds, however, little information exists on its impact on mosquitoes. Here we study how *Culex pipiens* mosquitoes transcriptionally respond to infection by two different *Plasmodium* species, *P. relictum* and *P. cathemerium*, differing in their virulence (mortality rate) and transmissibility (parasite presence in exposed mosquitoes' saliva). We studied the mosquito response to the infection at three critical stages of parasite development: the formation of ookinetes at 24 h post‐infection (hpi), the release of sporozoites into the hemocoel at 10 days post‐infection (dpi), and the storage of sporozoites in the salivary glands at 21 dpi. For each time point, we characterized the gene expression of mosquitoes infected with each *P. relictum* and *P. cathemerium* and mosquitoes fed on an uninfected bird and, subsequently, compared their transcriptomic responses. Differential gene expression analysis showed that most transcriptomic changes occurred during the early infection stage (24 hpi), especially when comparing *P. relictum* and *P. cathemerium‐*infected mosquitoes. Differentially expressed genes in mosquitoes infected with each species were related mainly to the metabolism of the immune response, trypsin, and other serine‐proteases. We conclude that these differences in response may partly play a role in the differential virulence and transmissibility previously observed between *P. relictum* and *P. cathemerium* in *Cx. pipiens*.

## 1 INTRODUCTION

Vector‐borne diseases are a major challenge for both human and animal health, representing more than 17% of emerging infectious diseases (WHO, 2021). Mosquitoes are vectors of relevant pathogens including viruses that cause yellow fever, dengue, or West Nile fever, parasites such as nematode worms which cause lymphatic filariasis, and haemosporidians, like *Plasmodium*, which cause malaria (Lehane, 2010). Malaria is one of the most important vector‐borne diseases for humans and, in 2020, it caused around 602,000 deaths (WHO, 2021). Malaria parasite species affect humans and related primates as well as other mammals, reptiles, or birds, driving some populations to extinction (van Riper et al., 1986).

Avian malaria is a worldwide distributed mosquito‐borne disease caused by *Plasmodium* parasites which use birds as obligate hosts (Valkiūnas, 2005). Avian *Plasmodium* is transmitted by mosquitoes, mainly of the genus *Culex*, which are the definitive hosts and where *Plasmodium* reproduces sexually (Valkiūnas, 2005). In birds, as a part of their life cycle, *Plasmodium* merozoites invade the host erythrocytes and can differentiate into mature gametocytes (Sinden, 1983). When a female mosquito feeds on a *Plasmodium*‐infected bird, gametocytes are released from the erythrocytes, and male and female gametes unite resulting first in the zygote, and then in the motile ookinete (Sinden, 2002). About 24 h post‐infection (hpi) the ookinete crosses the midgut epithelium and remains located between the epithelial surface and the basal lamina, where it transforms into a sessile oocyst. After about 10 days post‐infection (dpi), mature oocysts liberate thousands of sporozoites into the hemocoel. Sporozoites that survive the immune response of the mosquito eventually invade the salivary glands (Abraham & Jacobs‐Lorena, 2004; Sinden, 1983, 2002) from where they can be transmitted to a new avian host upon a mosquito bite.

For an appropriate development and transmission of the parasites, mosquitoes susceptible to the infection need to survive it, allowing parasites to complete their life cycle. The nature of the interaction between pathogens and mosquitoes determines the ability of a vector to acquire, maintain and transmit parasites to a new host, i.e. the vector competence (Beerntsen et al., 2000; Bonizzoni et al., 2013). Therefore, the vector competence is conditioned on the mosquito response against the pathogen (Higgs & Beaty, 2005), which includes several defence mechanisms. After a blood meal, mosquitoes synthesize and release serine proteases that constitute a chemical barrier against pathogens (Molina‐Cruz et al., 2005; Muller et al., 1995; Vizioli et al., 2001) and form a chitin‐containing peritrophic matrix around the blood. This matrix constitutes a physical barrier for harmful food particles, digestive enzymes, and pathogens (Lehane, 1997). Despite the existence of these and other barriers, some pathogens manage to reach the mosquito midgut, hemocoel, or internal organs and activate the immune response, which may be categorized into cellular and humoral immune response (Hillyer, 2016). The cellular response includes mechanisms such as phagocytosis, cellular encapsulation, autophagy, melanization, and induction of apoptosis, while the humoral response consists of the activation of signalling pathways that eventually result in the synthesis of factors with antimicrobial activity (Hillyer, 2016; Michel & Kafatos, 2005). In insects, one of the main immune signalling pathways is the Toll pathway, which is activated by the Spätzle cytokine and results in the activation of the transcription of antimicrobial peptides (AMPs) and other immune effectors that may combat pathogens (Kumar et al., 2018). In addition, two other pathways are involved in the response to *Plasmodium*, including avian malaria parasites, the immune deficiency (Imd) and the Janus Kinase signal transducer of activation (JAK–STAT) (Clayton et al., 2014; García‐Longoria et al., 2022).

The infection by *Plasmodium* and the mosquito response against infection ultimately result in a cost to vectors (Ahmed et al., 2002), which might depend, among other factors, on which *Plasmodium* species infects the mosquito. For example, Gutiérrez‐López et al. (2020), found a higher survival rate and transmissibility (measured as the presence of parasite DNA in the saliva of mosquitoes) in *Culex pipiens* infected with *Plasmodium cathemerium* compared to those infected with *Plasmodium relictum*. However, the genetic mechanisms that underlay these phenotypic differences are unknown. Part of the heterogeneity in parasites' virulence and therefore in the fitness consequences in their hosts (Gutiérrez‐López et al., 2020) is due to the remarkable cellular plasticity and transcriptional variation of *Plasmodium* (García‐Longoria et al., 2020). Avian *Plasmodium* is an extremely diverse clade with at least 55 species (Valkiūnas & Iezhova, 2018) divided into more than 1446 unique genetic lineages (Bensch et al., 2009). This extensive inter and intra‐specific genomic variation will translate into different phenotypic characters, including differences in virulence, which will also determine how the mosquitoes respond to the infection.

Although studies on avian malaria that use transcriptomic approaches have increased in the last few years, they have mainly focused on the avian host, addressing either the bird response to infection (e.g. Paxton et al., 2023; Videvall et al., 2015) or the gene expression of *Plasmodium* infecting birds (e.g. García‐Longoria et al., 2020; Videvall et al., 2017, 2021). In contrast, to the best of our knowledge, only three studies have analysed the gene expression of mosquitoes infected by avian *Plasmodium* (Zou et al., 2011; Ferreira et al., 2022; García‐Longoria et al., 2022; see Hernandez‐Caballero et al., 2023 for a recent review). However, none of them focus on *Cx. pipiens*, which is the main vector of avian *Plasmodium* in Europe. Another significant limitation is that all of these studies focus on *P. relictum* and, consequently, do not take into consideration the effect of the differences in virulence and transmissibility between *Plasmodium* species or lineages.

Here, we analyse the gene expression of *Cx. pipiens* infected by two widely distributed species of avian *Plasmodium* with different characteristics, namely *P. relictum* (lineage SGS1) and *P. cathemerium* (lineage PADOM02). To that end, we obtained RNA‐seq data of mosquitoes infected with the two *Plasmodium* species at three time points corresponding with key stages of parasite development in the vector: (1) during the formation of ookinetes, (2) during the release of sporozoites into the hemocoel, and (3) after sporozoites invade and are stored in the salivary glands. This study uses a natural avian malaria system that will help to address the current knowledge gaps on molecular mechanisms occurring during *Plasmodium* infections in mosquitoes. In addition, these results will allow us to further understand the differences in *Cx. pipiens* response against different *Plasmodium* species and how this might affect vector competence.

## 2 MATERIALS AND METHODS

### 2.1 Sampling and experimental conditions

We captured juvenile house sparrows (*Passer domesticus*) with mist nets in September 2020 at *Granja Escuela de Trigueros* (Huelva province, Spain). We ringed, weighed and measured the individuals before bringing them into captivity at the animal facilities of the Doñana Biological Station, following the ethical guidelines (article 34 RD 53/2013).

In the field, we took blood samples from each bird's jugular vein using a sterile syringe, taking both blood samples and blood smears. Blood samples were used to visually and molecularly identify the blood parasite infections and the parasite lineage identity. To do that, we extracted genomic DNA from blood samples using a Lithium Chloride protocol (Gemmell & Akiyama, 1996) and performed molecular amplification of *Plasmodium* cytochrome b gene to detect parasite infections following Hellgren et al. (2004). We sequenced the amplified products of the positive samples on both strands using Capillary Electrophoresis Sequencing by Macrogen (Madrid, Spain). We analysed the sequences using Geneious v. 2020.0.3 (Kearse et al., 2012) and identified lineages using MalAvi (Bensch et al., 2009). After molecular analyses, we chose three birds for further analyses, namely: A bird that was not infected by *Plasmodium*, *Haemoproteus* nor *Leucocytozoon* (control), a bird infected with *P. relictum* lineage SGS1, and a bird infected with *P. cathemerium* lineage PADOM02. To visually assess the intensity of parasitemia we analysed the blood smears of the selected birds infected by *P. cathemerium* and *P. relictum* using a light microscope Olympus CX33. The intensity of parasitemia was less than 1 parasite cell/15.000 erythrocytes in both cases.

We collected mosquito larvae in October 2020 in Aljaraque (Huelva province) and reared them following Gutiérrez‐López et al. (2020). We maintained larvae in dechlorinated water and fed them ad libitum with Hobby‐Mikrozell 20 mL/22 g (Dohse Aquaristik GmbH & Co.101 KG, D‐53501, Gelsdorf, Germany) and Hobby‐Liquizell 50 mL (Dohse Aquaristik GmbH & Co.101 KG, D‐53501, Gelsdorf, Germany). After emergence, we identified adults to the species level and sexed them following Gunay et al. (2018). We kept adult *Cx. pipiens* females in separate cages of 50 individuals maximum and fed them with a 10% sugar solution. We maintained both larvae and adult mosquitoes under controlled conditions (26°C ± 1, 55%–60% relative humidity (RH) and 12:12 light: dark photoperiod cycle).

We divided the 11‐day‐old adults (±1 day) into 3 groups mixing individuals originating from the different cages and allowed them to feed overnight on a *P. relictum*‐infected bird, a *P. cathemerium*‐infected bird and an uninfected control bird. Only one individual of each category was used in this experiment and they were exposed to mosquitoes only one time. In the morning after exposure, we separated the fed females of each group into three different cages and maintained them under the conditions described above.

For transcriptome analyses, we processed mosquitoes at three time points after exposure, 24 h post‐infection (hpi), 10 days post‐infection (dpi) and 21 dpi. At each time point, we created pools of 5 mosquitoes of each infection status capturing the mosquitoes alive and immediately transferring them to dry ice. To ensure that 24 h had passed since feeding, the degree of blood digestion was assessed according to the Sella scale, following Detinova et al. (1962), and selecting mosquitos classified as Sella stage IV. We preserved the mosquitoes at −80°C until RNA extractions were carried out. We collected a total of 36 samples including controls (4 pools × 3 time points × 3 conditions). Additionally, we recorded the mortality of each experimental group at 24 hpi, 10 and 21 dpi.

### 2.2 RNA extraction, library preparation, and sequencing

We extracted RNA and DNA from pools of 5 mosquitoes using TRIzol® (Invitrogen, Carlsbad, CA, USA) followed by column purification using RNeasy mini kit® (QIAGEN, Hilden, Germany) following Ferreira et al. (2022). We tested the remaining DNA for the presence of *Plasmodium* following Hellgren et al. (2004) confirming that parasite DNA was present in all positive samples and not present in negative samples. RNA samples were submitted to the Polo d'Innovazione di Genomica, Genetica e Biologia, Siena (Italy) where library preparation and sequencing were carried out. Briefly, RNA was quantified using the Qubit® 4.0 Fluorometer and RNA integrity was checked using the Fragment Analyser to measure the RNA Quality Number (RQN). Libraries were prepared following the QIAseqTM Stranded mRNA Selected Kit Handbook for Illumina Paired‐End Indexed Sequencing and sequenced in an Illumina NextSeq550 Flowcell, using the Illumina chemistry V2.5, 2 × 75 bp run.

### 2.3 Data analysis

To check the quality of the reads we used FastQC (Andrews, 2010) and MultiQC (Ewels et al., 2016). Then, we filtered low‐quality and under 36 bp reads using Trimmomatic (Bolger et al., 2014). Because the reference genome and annotations of *Cx. pipiens* are not published yet, we used the reference genome and annotations of phylogenetically closest species that were available in Ensembl, *Cx. quinquefasciatus* (https://metazoa.ensembl.org/Culex_quinquefasciatus/Info/Index). We used STAR (Dobin et al., 2012) to map the short reads to the reference genome of *Cx. quinquefasciatus* and RSEM (Li & Dewey, 2011) to quantify gene abundances (see further details in Supplementary Material S1).

The following steps were performed in R (R Core Team, 2021) using Bioconductor packages (Huber et al., 2015). We carried out a Variance Stabilizing Transformation (VST) of the counts to represent the samples on a PCA plot. Then, we used the DESeq2 package (Love et al., 2014) to perform the differential gene expression analysis comparing: (i) *P. relictum*‐infected mosquitoes vs. controls, (ii) *P. cathemerium*‐infected mosquitoes vs. controls, and (iii) *P. relictum‐*infected mosquitoes vs. *P. cathemerium*‐infected mosquitoes. We kept those genes with an adjusted *p*‐value <0.01 and sorted them by the Log2 Fold change (LFC) estimations, considering genes with a LFC > 1 or LFC < −1, excepting the gene CPIJ006792 due to its relevance in mosquito immune response (Shin et al., 2006). We finished the differential gene expression analysis visualizing differentially expressed genes by MA plots and an UpSet plot. Additionally, we performed a second differential gene expression analysis to quantify differences in the responses of mosquitoes among the three time points.

Finally, we carried out a Gene Ontology (GO) enrichment analysis for the up and down‐regulated genes (without filtering for LFC) using the topGO package (Alexa & Rahnenfuhrer, 2010) including the ‘Biological Process’ and ‘Molecular Function’ categories from VectorBase (Giraldo‐Calderón et al., 2015). For the enrichment analysis, we used classical algorithms and Fisher's exact tests and considered enriched the GO terms with *p*‐value <0.01. We used the ggplot2 (Wickham, 2016) package to visualize the enriched GO terms as described by Bonnot et al. (2019). To simplify the graphical representation, we removed the redundant GO terms using REVIGO (http://revigo.irb.hr/).

## 3 RESULTS

At the end of the experiment, higher mortalities were found for mosquitoes exposed to *P. relictum*‐infected birds (9.17%) compared to those exposed to *P. cathemerium*‐infected birds (5.63%) and control birds (6.35%) (see Table S2).

### 3.1 Transcriptomics data description

We obtained 36 mRNA‐seq libraries: four replicates for three infection statuses at three time points. The mean number of reads per library was 33,177,082, ranging from 15,406,828 to 45,332,626 reads for raw samples. Raw data are publicly available at the European Nucleotide Archive ENA database (https://www.ebi.ac.uk/ena/browser/home) under project accession number PRJEB41609, Study ERP125411. The percentage of reads kept after trimming ranged from 80.56% to 99.52%, (12,411,723 to 44,887,231 reads; see Table S1). The MultiQC report showed a mean quality score above q30 in all base calls across the read. On average, 80.87% of reads mapped to the genome of *Cx. quinquefasciatus*. For downstream analyses, we removed one control sample and one *P. cathemerium*‐infected sample taken at 24 hpi that had barcoding errors due to laboratory processing, leaving three samples for these two treatments.

### 3.2 Early transcriptomic response to infection

**FIGURE 1:**
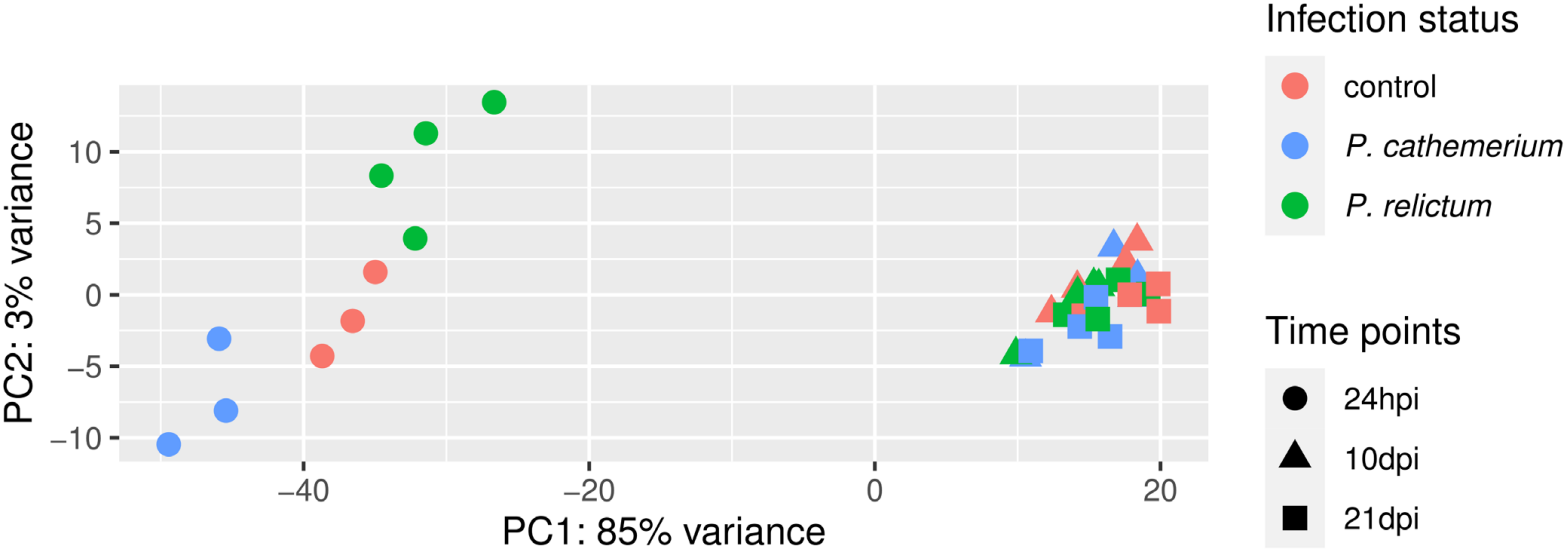
Principal component analysis (PCA) of transcriptome variation *in Cx. pipiens* at three time points after feeding on either an uninfected bird (control), a *P. relictum*‐infected bird and a *P. cathemerium*‐infected bird. Time points analysed were 24 h post‐infection (hpi), 10 days post‐infection (dpi) and 21 dpi. The x‐axis shows the first principal component score, which captures 85% of the variation, and the y‐axis shows the second principal component score, which captures 3% of the variation.

The principal component analysis (PCA) results show that most of the transcriptome variation was contained in the PC1 (85% var.) driven by the time post‐bloodmeal, for both *Plasmodium* species infections. At 24 hpi, there were clear differences (PC2, 3% var.) between control samples, *P. cathemerium*, and *P. relictum*‐infected mosquitoes. By contrast, there were no clear transcriptomic differences at later stages, such as between 10 and at 21 dpi, nor between infection statuses (Figure 1).

In addition, when analysing differences in gene expression between the three time points, we found 6125 genes differentially expressed between 24 hpi and 10 dpi and 6422 between 24 hpi and 21 dpi, with 5436 genes coinciding in both contrasts (Figure S1). Only 28 genes were differentially expressed between 10 and 21 dpi. The enriched GO terms for the up‐regulated genes were mostly related to biological processes of biomolecule metabolism (Figure S2) and molecular functions such as peptidase and hydrolase activities (Figure S3) (see Supplementary Material S2 for further details).

### 3.3 Differential gene expression analysis and enrichment analysis unveil the clues of the mosquito transcriptomic response to *Plasmodium* infections

**FIGURE 2:**
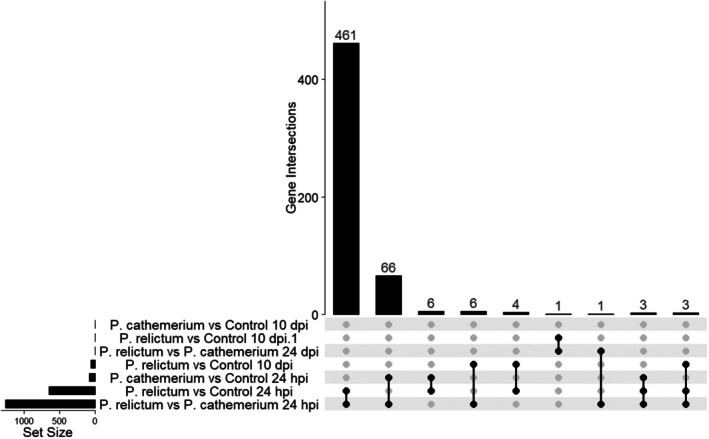
UpSet plot showing overlap size of sets of differentially expressed genes for (i) *P. relictum*‐infected mosquitoes vs. controls, (ii) *P. cathemerium‐*infected mosquitoes vs. controls, and (iii) *P. relictum*‐infected mosquitoes vs. *P. cathemerium*‐infected mosquitoes at three time points (24 hpi, 10 and 21 dpi). The top vertical bar plot shows the number of genes (y‐axis) contained in each intersection (x‐axis). The horizontal bar plot at the bottom shows the number of differentially expressed genes for each comparison.

Overall, 2051 genes were differentially expressed in *Cx. pipiens*, from which 572 showed a LFC higher than 1 or lower than −1 (see Table 1 for differentially expressed genes distribution among comparisons and Supplementary Material S2 for the full list of differentially expressed genes). Most transcriptomic differences were found at an early infection stage (24 hpi), especially when comparing *P. relictum*‐infected mosquitoes vs. *P. cathemerium‐*infected mosquitoes, and *P. relictum‐*infected mosquitoes vs. controls. No differences were found between mosquitoes infected by *P. cathemerium* and those infected by *P. relictum* at 10 dpi, or between mosquitoes infected with *P. relictum* and controls at 21 dpi. At 24 hpi, the comparisons *P. relictum*‐infected mosquitoes vs. controls and *P. relictum* vs. *P. cathemerium*‐infected mosquitoes shared 461 differentially expressed genes (Figure 2; see Supplementary Material S3 for MA plots).

**TABLE 1:** The number of differentially expressed genes (DEG) with a *p* < .01 for all comparisons at 24 h post‐infection (hpi), 10 days post‐infection (dpi), and 21 dpi. In brackets, DEG filtered by log2 fold change (LFC) > 1 for up‐ regulated genes and LFC < −1 for down‐regulated genes. No DEG were found for the comparisons *P. relictum* vs. *P. cathemerium* infected mosquitoes at 10 dpi and *P. cathemerium* infected mosquitoes vs. controls at 21 dpi.

Time‐point	Comparisons	Total DGE	Up‐regulated	Down‐regulated
24 hpi	*P. relictum* vs. controls	645 (115)	107 (16)	538 (99)
	*P. cathemerium* vs. controls	83 (21)	79 (19)	4 (2)
	*P. relictum* vs. *P. cathemerium*	1256 (406)	330 (49)	926 (357)
10 dpi	*P. relictum* vs. controls	62 (26)	46 (24)	16 (2)
	*P. cathemerium* vs. controls	1 (0)	1 (0)	0 (0)
21 dpi	*P. relictum* vs. controls	2 (2)	1 (1)	1 (1)
	*P. relictum* vs. *P. cathemerium*	2 (2)	1 (1)	1 (1)

#### 3.3.1 *Cx. pipiens* gene expression response to *P. relictum* infection

At 24 hpi, up‐regulated genes in *P. relictum*‐infected *Cx. pipiens* compared to controls included a cecropin gene (CPIJ005108), which is part of the Toll pathway in the immune response, and a mitochondrial NADH–ubiquinone oxidoreductase gene (CPIJ009076). Another gene involved in the Toll pathway, *spätzle 1B* (CPIJ006792), was also up‐regulated in *P. relictum*‐infected mosquitoes, although with a LFC = 0.3984. Within the down‐regulated genes there were four multicopper oxidase genes (CPIJ016802, CPIJ010466, CPIJ012244, and CPIJ010465), four chymotrypsin (CPIJ003915, CPIJ018205, CPIJ007838, and CPIJ006568) and two serine protease genes (CPIJ004984 and CPIJ002112) (Figure S4A).

At 10 dpi, when compared to controls, mosquitoes infected by *P. relictum* up‐regulated two ribosomal genes (CPIJ040837 and CPIJ009519). At 21 dpi, the aromatic amino acid decarboxylase gene (CPIJ010562) was down‐regulated (Figure S4B).

**FIGURE 3:**
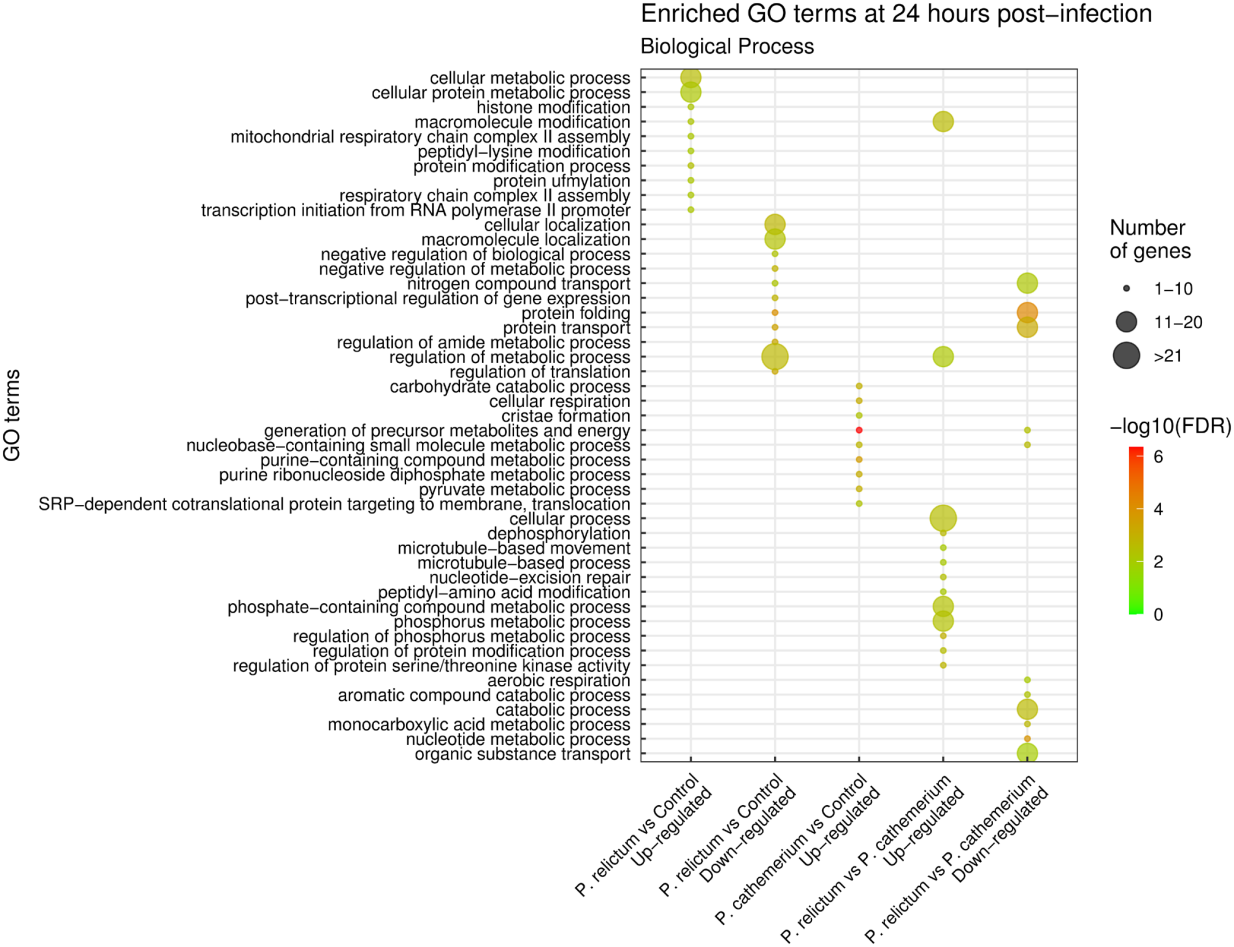
Dot plot of significantly (adjusted *p*‐value <0.01) enriched GO biological processes for differentially expressed genes (up and down‐regulated) at 24 hpi for (i) *P. relictum*‐infected mosquitoes vs. controls, (ii) *P. cathemerium*‐infected mosquitoes vs. controls, and (iii) *P. relictum*‐infected mosquitoes vs. *P. cathemerium‐*infected mosquitoes. We did not find enriched GO terms for down‐regulated genes for *P. cathemerium* infected mosquitoes vs. controls. Larger dots correspond to a higher number of significant genes, and the colour gradient goes from green for the least significant terms to red for the most significant terms, measured as False Discovery Rate (FDR).

**FIGURE 4:**
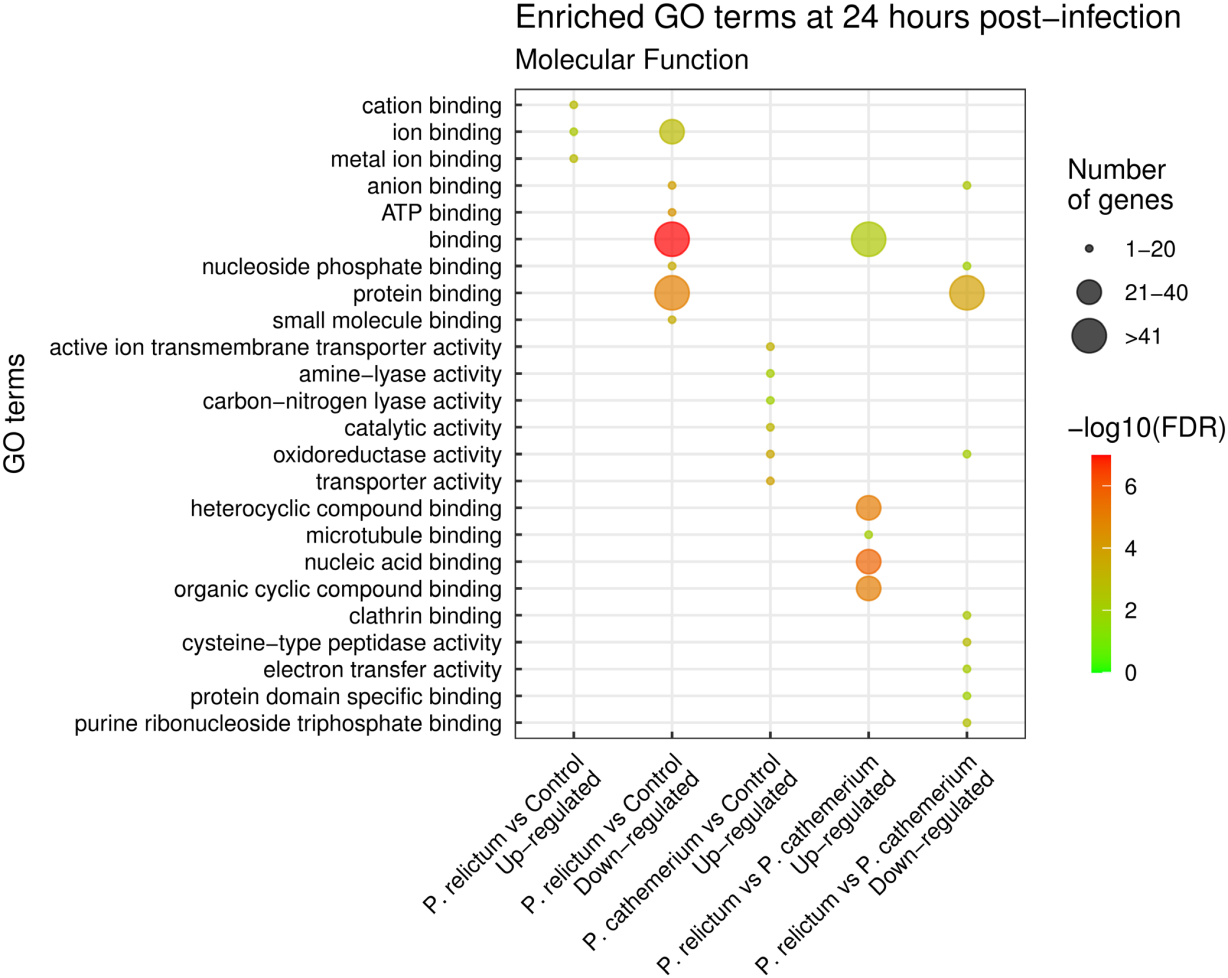
Dot plot of significantly (adjusted *p*‐value <0.01) enriched GO molecular functions for differentially expressed genes (up and down‐regulated) at 24 hpi for (i) *P. relictum*‐infected mosquitoes vs. controls, (ii) *P. cathemerium* infected mosquitoes vs. controls, and (iii) *P. relictum*‐infected mosquitoes vs. *P. cathemerium*‐infected mosquitoes. We did not find enriched GO terms for down‐regulated genes for *P. cathemerium* infected mosquitoes vs. controls.

At 24 hpi, the GO terms within the Biological Process category related to cellular mechanisms and mitochondrial chain complex assemblies presented the greatest number of up‐regulated genes. Within down‐regulated genes, the enriched biological processes included molecule metabolism, transport and localization (Figure 3; Supplementary Material S3). The molecular functions enriched for up‐regulated genes were cation binding, metal ion binding and ion binding. Protein, ATP, and nucleic acids binding molecular functions (including purine ribonucleoside triphosphate, purine nucleotide, purine ribonucleotide, adenyl nucleotide and adenyl ribonucleotide, among others) were enriched for down‐regulated genes (Figure 4; Supplementary Material S4).

At 10 dpi enriched biological processes were related to amino acid biosynthesis for up‐regulated genes and to protein geranylgeranylation for down‐regulated genes (Supplementary Material S3). Molecular functions included actin monomer binding and GTPase activity for up‐regulated genes (Supplementary Material S4).

#### 3.3.2 *Cx. pipiens* gene expression during response to *P. cathemerium* infection

In *P. cathemerium*‐infected mosquitoes compared to controls at 24 hpi (Figure S5), we found up‐regulated genes related to digestive enzymes like *trypsin 2 precursor* (CPIJ005273), two protein G12 precursors (CPIJ012846 and CPIJ012848), and genes related to the mitochondrial electron chain including genes for an ADP‐ATP carrier protein (CPIJ005941), a mitochondrial phosphate carrier protein (CPIJ013141), and the cytochrome P450 4C51 (CPIJ018944). The *pom1* gene (CPIJ019938) was down‐regulated. At both 10 and 21 dpi no genes presented differential expression with |LFC| > 1.

At 24 hpi, GO terms associated with biological processes were mostly related to the generation of precursor metabolites and energy, ATP generation, and nucleic acids metabolic processes, including nucleoside phosphate, ribose phosphate, nucleotide, ribonucleotide, purine nucleotide and purine ribonucleotide metabolic processes (Figure 3; Supplementary Material S3). GO terms associated with molecular functions included ion and transmembrane transporter, oxidoreductase and proton‐transporting ATP synthase activities (Figure 4; Supplementary Material S4).

#### 3.3.3 *Cx. pipiens* respond differently to *P. relictum* and *P. cathemerium* infections

At 24 hpi, the comparison *P. relictum‐* vs. *P. cathemerium‐*infected mosquitoes showed the largest number of differentially expressed genes (Table 1; Figure S6). The up‐regulated genes included four zinc finger protein genes (CPIJ015530, CPIJ012592, CPIJ008782, and CPIJ009347). On the other hand, mosquitoes infected by *P. relictum* compared to those infected by *P. cathemerium* showed down‐regulated genes of five protein G12 precursors (CPIJ005176, CPIJ012848, CPIJ012844, CPIJ012845, and CPIJ012846), four multicopper oxidases (CPIJ016802, CPIJ010466, CPIJ010465, and CPIJ000864), two chitin synthases (CPIJ014268 and CPIJ014269), a peritrophic membrane chitin binding protein (CPIJ007042), and a number of serine proteases including five chymotrypsin genes (CPIJ003915, CPIJ018205, CPIJ007838, CPIJ006542, and CPIJ006568) and three trypsin precursors (CPIJ005273, CPIJ006019, and CPIJ004660). From the down‐regulated genes, three multicopper oxidases and three chymotrypsin precursors were also down‐regulated in *P. relictum*‐infected mosquitoes compared to controls, and two G12 protein precursors and one trypsin precursor were also up‐regulated in *P. cathemerium*‐infected mosquitoes.

No differentially expressed genes were found at 10 dpi and only 2 genes at 21 dpi, a down‐regulated testicular acid phosphatase precursor (CPIJ007697) and an up‐regulated uncharacterized protein.

At 24 hpi, biological processes related to metabolic regulation were enriched for up‐regulated genes, while down‐regulated genes included nucleic acid nucleotide metabolic processes (Figure 3; Supplementary Material S3), some of them also enriched for up‐regulated genes *in P. cathemerium*‐infected mosquitoes compared to controls. Molecular functions like microtubule and nucleic acid binding were enriched in up‐regulated genes in mosquitoes infected by *P. relictum* compared to those infected by *P. cathemerium*. Most down‐regulated enriched molecular functions were related to protein and nucleotide binding (Figure 4; Supplementary Material S4), as observed in mosquitoes infected by *P. relictum* compared to controls.

## 4 DISCUSSION

How mosquitoes respond to infection and the impact of such infection will ultimately influence the transmission of different *Plasmodium* species. For example, *P. relictum* and *P. cathemerium* infections in *Cx. pipiens* have a different impact on the mosquitoes’ fitness. Gutiérrez‐López et al. (2020) reported that *Cx. pipiens* exposed to *P. relictum* suffered higher mortality and had lower transmissibility than those exposed to *P. cathemerium*. But little is known about the genetic underpinnings of mosquito response to *Plasmodium* infections. Here, we compare for the first time the transcriptional response of *Cx. pipiens* infected by these two avian *Plasmodium* species. Although our study was not designed to carry out a survival analysis, we found a similar trend than the observed in Gutiérrez‐López et al. (2020), with a higher apparent incidence of mortality in *P. relictum‐* than in *P. cathemerium‐*infected mosquitoes (9.17% vs. 5.63%). Regarding the transcriptional response, our results show that although responses of infected *Cx. pipiens* by these *Plasmodium* species share some common pathways, there are key differences in gene expression associated with the immune response and serine‐protease synthesis. This information provides new insights into the molecular mechanism underlying *Cx. pipiens* immune response against *Plasmodium*. Furthermore, by comparing two *Plasmodium* species, we show that there is variation in the immune response of *Cx. pipiens* to different avian malaria species. This knowledge will in the long term help to understand the complex interaction between the mosquitos and different malaria parasites and potentially uncover the key to understanding differences in transmission and fitness consequences for the mosquitoes.

### 4.1 Early and different responses to the infections

Blood feeding triggers a strong early response in female mosquitoes because they have to digest the blood to obtain nutrients needed for egg development (Dana et al., 2005; de Swart et al., 2023; Nag et al., 2021). In our study, mosquitoes from all treatments, including controls, showed a high response 24 h after blood feeding compared to 10 and 21 dpi, with up‐regulated genes involved in biomolecule metabolism biological processes and molecular functions such as peptidase and hydrolase activities. This is consistent with previous studies where an up‐regulation of genes related to peptidase, oxidoreductase, and hydrolase activities (Nag et al., 2021) and metabolism (Sanders et al., 2003) were recorded. After blood ingestion, the mosquito immune response is also activated (Luckhart et al., 2003; Nag et al., 2021), which is further activated when exposed to avian *Plasmodium‐*infected blood meals (García‐Longoria et al., 2022).

We found that the vast majority of the differentially expressed genes between *Plasmodium‐*infected and uninfected mosquitoes were also found at 24 hpi. Between 18–24 h after blood feeding ookinetes form, invade the peritrophic matrix leaving behind the blood bolus and start the midgut epithelium cell invasion (Baia‐da‐Silva et al., 2018; Cirimotich et al., 2010; Valkiūnas et al., 2015). Ookinete formation and invasion of the midgut epithelium are considered critical steps that will determine the success of the infection. Parasite abundance drops drastically during this step due to lumenal and epithelial immune responses mounted by the mosquito (Cirimotich et al., 2010), which may explain the significant differences in gene expression between uninfected and infected mosquitoes at 24 hpi (Ferreira et al., 2022). In addition, Vlachou et al. (2005) using the *Plasmodium berghei* ‐ *Anopheles gambiae* model found that 7% of the mosquito transcriptome was differentially regulated during ookinete invasion of the midgut.

At 24 hpi, for both mosquitoes infected with *P. cathemerium* and *P. relictum*, we found an increase in differentially expressed genes and enriched GO terms related to the mitochondrial respiratory chain activity that ultimately produces reactive oxygen species (ROS) (Kowaltowski et al., 2009). Small regulatory changes in the mitochondrial respiratory chain can drastically affect ROS generation (Korshunov et al., 1997; Kowaltowski et al., 2009). For example, *Anopheles stephensi* and *An. gambiae* increased the levels of ROS in response to *Plasmodium* infection (Han, 2000; Kumar et al., 2003), and higher levels of ROS improve mosquito survival after a bacterial infection (Molina‐Cruz et al., 2008). At 24 hpi we also found most of the differences in gene expression between mosquitoes infected by each of these parasites.

### 4.2 Differential immune response between mosquitoes exposed to *P. relictum* or *P. cathemerium*

We found important differences in the expression of genes associated with the immune response between *P. relictum* and *P. cathemerium*‐infected mosquitoes. At 24 hpi, in *Cx. pipiens* infected by *P. cathemerium* compared to controls, we found that two of the most up‐regulated genes were protein G12 precursors. These two genes and other three protein G12 precursor genes were down‐regulated in mosquitoes infected by *P. relictum* compared to those infected by *P. cathemerium*. G12 transcripts accumulate in the midgut of mosquitoes after blood feeding (Bonizzoni et al., 2012; Shao et al., 2005), and may aid in erythrocyte digestion given their hemolytic activity (Foo et al., 2021). G12 transcripts have been suggested to play a role in immune function because (i) G12 protein in *Aedes aegypti* has a high level of identity with cockroach allergens (Morlais et al., 2003), (ii) it is up‐regulated after flavivirus infection via the JAK–STAT pathway (Etebari et al., 2017), and (iii) it has a cytolytic effect on flaviviruses and several types of eukaryotic cells (Foo et al., 2021). In fact, a G12 protein gene was found up‐regulated in *Ae. aegypti* 12 h after feeding on blood infected with *Plasmodium gallinaceum* (Morlais et al., 2003), suggesting a potential role in the immune response against these parasites as well.

In *P. relictum*‐infected mosquitoes two of the up‐regulated genes at 24 hpi were related to the Toll pathway of the innate immune response when compared to the controls: The genes encoding Spätzle and Cecropin N proteins. In insects, when a pathogen is recognized, the extracellular Spätzle cytokine activates the Toll receptors, which regulate the antimicrobial peptides (AMPs), an essential innate immune response (De Gregorio, 2002; Shia et al., 2009). The AMPs eventually kill pathogens by a number of strategies including disrupting the microbial membrane (Shen et al., 2018). Spätzle protein activates a Toll receptor in *Ae. aegypti* mosquitoes when infected with the fungus *Beauveria bassiana* (Shin et al., 2006) and in other insects after bacterial and fungal exposure (Bae et al., 2021). Cecropins are one of the largest groups of insect AMPs found in the orders Diptera, Lepidoptera, and Coleoptera, among others (An et al., 2009; Memarpoor‐Yazdi et al., 2013; Vizioli et al., 2000). The activation of the Toll pathway and specifically cecropin‐analogs may kill *Plasmodium* parasites (Frolet et al., 2006; Jaynes et al., 1988) and disrupt sporogonic development by aborting the normal development of oocysts (Gwadz et al., 1989; Kim et al., 2004), which could reduce the parasite load in posterior infection stages. Our results showing the role of the Toll pathway are consistent with a recent study addressing the effect of *P. relictum* (lineage SGS1) on the immune system of *Cx. quinquefasciatus* (García‐Longoria et al., 2022). They found that over 50% of immune genes identified as being part of the Toll pathway in *Cx. quinquefasciatus* were up‐regulated after exposure to *P. relictum*.

### 4.3 Differential expression of genes associated with trypsin and serine metabolism

Serine proteases have several functions in insects including blood digestion (Borovsky & Schlein, 1988) and mediation in the immune response (melanization, cytokine activation, and antimicrobial peptides; Jiang et al., 2010). Trypsins and chymotrypsins, two types of serine proteases, are two essential digestive enzymes in mosquitoes (Borges‐Veloso et al., 2012; Molina‐Cruz et al., 2005). The synthesis of trypsins and chymotrypsins is triggered after mosquito blood feeding to digest the chitin‐containing peritrophic matrix (Muller et al., 1995; Vizioli et al., 2001) which is fully formed at 24 h post‐feeding (Hegedus et al., 2009). However, the production of trypsin and chymotrypsin may be down or up‐regulated by the infection with different parasites (Borovsky & Schlein, 1987; Serrano‐Pinto et al., 2010; Shahabuddin et al., 1996). Mosquito trypsin may be a signal for *Plasmodium* ookinetes to cross the peritrophic matrix at the right time for proper *Plasmodium* development (Shahabuddin et al., 1996). In particular, mosquito trypsin proteases play a fundamental role in allowing *P. gallinaceum* to cross the peritrophic matrix by activating a *Plasmodium* prochitinase enzyme (Shahabuddin et al., 1996). In fact, trypsin inhibitors block the development of *Plasmodium* oocysts (Shahabuddin et al., 1996), suggesting that mosquito trypsin is a key molecule for pathogen infection.

When compared to controls, mosquitoes exposed to *P. cathemerium* up‐regulated the trypsin 2 precursor gene, while mosquitoes exposed to *P. relictum* strongly down‐regulated genes of chymotrypsins and serine protease precursors. Furthermore, when comparing gene expression between *P. relictum*‐ and *P. cathemerium*‐infected mosquitoes, we found that mosquitoes exposed to *P. relictum* down‐regulated the same chymotrypsins and serine protease precursors that they did when compared to controls. In addition, the mosquitoes exposed to *P. relictum* also downregulated  other chymotrypsins, serine protease precursors, and three trypsin precursors. This differential expression was found at 24 hpi, therefore coinciding with the moment when the peritrophic matrix is fully formed and the ookinetes are crossing it. Agreeing with the findings of Shahabuddin et al. (1996) for the infection by *P. gallinaceum*, we also found that trypsin (*trypsin 2 precursor*) was up‐regulated in mosquitoes infected with *P. cathemerium* when compared with uninfected mosquitoes. This suggests the potential role of the trypsin proteases in allowing *P. cathemerium* to cross the peritrophic matrix. In contrast, mosquitoes infected with *P. relictum* had several down‐regulated genes characterized as serine proteases, including several chymotrypsin and one trypsin. Ferreira et al. (2022) also found serine‐type endopeptidase activity enriched for down‐regulated genes when studying the expression of *Cx. quinquefasciatus* infected by *P. relictum*. We hypothesize that the lower levels of trypsin in response to *P. relictum* infection might lead to a lower number of oocysts and would explain why the transmissibility of *P. relictum* is lower than that of *P. cathemerium* (Gutiérrez‐López et al., 2020).

In addition, an under‐expression of serine‐proteases related to blood digestion such as chymotrypsins could partly block the digestion of the blood, which is very rich in proteins (Kumar et al., 2017). This would make it difficult to obtain nutrients needed for various processes like egg formation and could lead to digestive dysregulation in the mosquito which might increase mortality. Here mosquitoes seem to be down‐regulating important processes due to the infection with *P. relictum* which might have a cost for the mosquito, induce damage and decrease the tolerance to infection.

### 4.4 Reduced differential gene expression at 10 and 21 days post‐infection

An unexpected result was the strong decrease of differential gene expression between infected and uninfected mosquitoes at 10 dpi. We chose this time point because at about 10 dpi *Plasmodium* mature oocysts release the sporozoites into the mosquito hemocoel (Aly et al., 2020). However, at this time point, only *P. relictum*‐infected mosquitoes compared to controls showed differences in gene expression and the number of differentially expressed genes decreased significantly compared to 24 hpi. Similar results were obtained by Ferreira et al. (2022) when studying gene expression of *Cx. quinqueafasciatus* exposed to *P. relictum*. In addition, the absence of differential expression in mosquitoes exposed to *P. relictum* compared to those exposed to *P. cathemerium* at 10 dpi may be due to differences in the developmental time of different species of *Plasmodium* (Sinden, 1983). *P. cathemerium* produces sporozoites faster than *P. relictum* when infecting *Cx. pipiens* mosquitoes (Aly et al., 2020; Kazlauskienė et al., 2013), and therefore they may not be exactly at the same point of development within the mosquito at 10 dpi. In addition, when the sporozoites are released, part of them are destroyed by a rapid immune response (Hillyer et al., 2007), which may also result in a lower genic response by the mosquito.

By 21 dpi, the differences in gene expression were drastically reduced, with only two genes differentially expressed in mosquitoes infected with *P. relictum*, and none in those infected with *P. cathemerium*. This decrease in differential expression towards the end of the infection has been found before in *Cx. quinqueafasciatus* infected with *P. relictum* (Ferreira et al., 2022; García‐Longoria et al., 2022). Although the causes behind this decrease are not clear, we hypothesize that the response towards the end of the infection might be localized to the salivary glands, and by analysing whole mosquitoes we are diluting any potential effect.

Furthermore, the probability of infection following parasite ingestion is not 100% (e.g. Gutiérrez‐López et al., 2020) and depends on the success of the ookinetes crossing the midgut epithelium, which happens around 24 h post‐feeding. This results in a certain intra‐pool variability that may result in a lack of differential gene expression in mosquitoes sampled at 10 and 21 dpi.

### 4.5 Comparisons across avian malaria studies

Although studies analysing the response of mosquitoes to avian malaria are still scarce we can already see some common patterns and differences. Two other recent studies have analysed the response to *P. relictum* infection in *Cx. quinquefasciatus*, a species closely related to *Cx. pipiens*. A common pattern found in all studies is the progressive reduction in the differential gene expression at 10 and 21 dpi when compared to 24 hpi. In addition, like García‐Longoria et al. (2022), we found the activation of the Toll‐like receptor pathway in response to *P. relictum* SGS1 infection. Interestingly, Ferreira et al. (2022) did not find this pattern. Although different factors may be influencing this result, like experimental procedures or the genetic differences between the populations of *Cx*. *quinquefasciatus* used, one of the main factors might have been the lineage used for the infection. Ferreira et al. (2022) infected the mosquitoes with another lineage, *P. relictum* GRW4, which dominates in America. Like Ferreira et al. (2022), the response to infection we found for mosquitoes infected with *P. cathemerium*, was different. But, a similar pattern has been found before. Shahabuddin et al. (1996) reported the important role of a mosquito trypsin in the passage of *P. gallinaceum* through the peritrophic matrix, as our results indicate for *P. cathemerium*. Altogether these results suggest that different *Plasmodium* species or lineages may trigger a differential immune response in mosquitoes.

### 4.6 Limitations of this study

Due to the elevated cost of the analyses and the limitations of working with natural populations of both hosts and mosquitoes, we used four biological replicates (three in two treatments due to a laboratory error) for each experimental group and time point. This sample size is similar to those used in previous RNA‐seq studies (Todd et al., 2016), including those focused on the effects of avian *Plasmodium* infections in mosquitoes (e.g. García‐Longoria et al. 2022). However, when using wild populations that often have high biological variance, smaller sample sizes may increase the probability of confounding biological differences with real differences between experimental groups (Todd et al., 2016). In this regard, we worked with pools of individuals (*n* = 5), which reduced the impact of individual samples when working with samples that may have high biological variation (Kendziorski et al., 2005).

The application of the sample size formula used by RNASeqPower R package (Therneau et al., 2023) revealed that, for our dataset, the sample size required to detect genes with |LFC = 1| (i.e. variation of 100%) with statistical power between 80%–90% is 4–6 replicas per treatment, which is within the range used here. However, to detect genes with smaller effects we would need more replicas. For example, to detect a variation of 50% we would need 13–16 replicas (Table S3). Although this suggests that differences in gene expression within this range of variation might be under identified in this study, we have been able to identify the major pathways activated during the immune response, which are evolutionary conserved across insect taxa and usually have strong effects (Viljakainen, 2015).

### 4.7 Concluding remarks

Because we used naturally infected wild birds as *P. cathemerium* and *P. relictum* donors and wild collected mosquitoes, the results obtained here represent a good example of a natural system. In this respect, we found a different transcriptomic response to infections, especially at 24 hpi. This time point coincides with one of the key stages of *Plasmodium* development in mosquitos, when the ookinetes form, cross the peritrophic matrix and start to invade the midgut epithelium. Both *Plasmodium* species elicit an innate immune response, with mosquitoes exposed to *P. cathemerium* up‐regulating the antimicrobial G12 proteins and those exposed to *P. relictum* activating the Toll Pathway (Spätzle and Cecropin N proteins), which may result in a reduction of *Plasmodium* development (Frolet et al., 2006). On the other hand, the lower levels of trypsin in mosquitoes exposed to *P. relictum* compared to those exposed to *P. cathemerium* may affect the parasite development within the mosquito, which may affect parasite transmission (Gutiérrez‐López et al., 2020). If the cost of this response is high, this can also potentially lead to higher mosquito mortality. In particular, the proteases and trypsins are necessary to digest the blood meal, and if levels are too low this might increase mosquito mortality. Future studies are necessary to better understand how the observed differences are linked to differences in transmission. In addition, it will also be important to understand how these differences may be related to the different ecology and incidence of *Plasmodium* lineages/species in the wild.

## AUTHOR CONTRIBUTIONS

María José Ruiz‐López, Josué Martínez‐de la Puente and Jordi Figuerola Borras designed the original study. María José Ruiz‐López developed the experimental assay and the laboratory work. Marta Garrigós did the bioinformatic work and interpreted the results with María José Ruiz‐López and Guillem Ylla. Marta Garrigós wrote the first original draft of the manuscript and subsequent versions with considerable assistance from the rest of the authors.

## FUNDING INFORMATION

This publication was supported by the project Research Infrastructures for the control of vector‐borne diseases (Infravec2; project number 6738), which has received funding from the European Union's Horizon 2020 research and innovation programme, under grant agreement No 731060; project PGC2018‐095704‐B‐I00 from Agencia Española de Investigación supported by FEDER Funds from the European Union and the computing infrastructure provided by ICTS‐RBD‐CSIC. This study was also partially financed by the PID2020‐118205GB‐I00 grant to JMP funded by MCIN/AEI/10.13039/501100011033. MG was supported by a FPI grant (PRE2021‐098544). GY contributions were supported by the Faculty of Biochemistry, Biophysics and Biotechnology at Jagiellonian University (Poland), under the Strategic Programme Excellence the Polo d'Innovazione di Genomica, Genetica e Biologia, Siena (Italy) for the samples Initiative. Funding for open access charge: Universidad de Granada/CBUA.

## CONFLICT OF INTEREST STATEMENT

The authors declare that they have no conflicts of interest.

## Supporting information


Supplementary Material S1.

Supplementary Material S2.

Supplementary Material S3.

Supplementary Material S4.

## Data Availability

Raw sequences generated in this study have been submitted to the European Nucleotide Archive ENA database (https://www.ebi.ac.uk/ena/browser/home) under project accession number PRJEB41609, Study ERP125411. Sample metadata are available at https://doi.org/10.20350/digitalCSIC/15708.

## References

[mec17240-bib-0001] Abraham, E. , & Jacobs‐Lorena, M. (2004). Mosquito midgut barriers to malaria parasite development. Insect Biochemistry and Molecular Biology, 34(7), 667–671. 15242707 10.1016/j.ibmb.2004.03.019

[mec17240-bib-0002] Ahmed, A. M. , Baggott, S. L. , Maingon, R. , & Hurd, H. (2002). The costs of mounting an immune response are reflected in the reproductive fitness of the mosquito *Anopheles gambiae* . Oikos (Copenhagen, Denmark), 97(3), 371–377. 10.1034/j.1600-0706.2002.970307.x

[mec17240-bib-0003] Alexa, A. , & Rahnenfuhrer, J. (2010). topGO: Enrichment analysis for gene ontology. R Package Version, 2(0), 2010.

[mec17240-bib-0004] Aly, M. Z. Y. , Mohamed, I. I. I. , Sebak, S. I. , Vanstreels, R. E. T. , & El Gendy, A. M. (2020). Morphological and molecular characterization of *plasmodium cathemerium* (lineage PADOM02) from the sparrow *Passer domesticus* with complete sporogony in *Culex pipiens* complex. Parasitology, 147(9), 985–993. 10.1017/S0031182020000566 32338240 PMC10317643

[mec17240-bib-0005] An, C. , Jiang, H. , & Kanost, M. (2009). Proteolytic activation and function of the cytokine Spätzle in the innate immune response of a lepidopteran insect, Manduca Sexta. The FEBS Journal, 277(1), 148–162. 19968713 10.1111/j.1742-4658.2009.07465.x PMC2805777

[mec17240-bib-0006] Andrews, S. (2010). *FastQC: A quality control tool for high throughput sequence data* [online].

[mec17240-bib-0007] Bae, Y. , Jo, Y. , Patnaik, B. , Kim, B. , Park, K. , Edosa, T. , Keshavarz, M. , Kojour, M. A. M. , Lee, Y. S. , & Han, Y. S. (2021). *Tenebrio molitor* Spätzle 1b is required to confer antibacterial defense against gram‐negative bacteria by regulation of antimicrobial peptides. Frontiers in Physiology, 12, 758859. 10.3389/fphys.2021.758859 34867464 PMC8637286

[mec17240-bib-0008] Baia‐da‐Silva, D. C. , Alvarez, L. C. S. , Lizcano, O. V. , Costa, F. T. M. , Lopes, S. C. P. , Orfanó, A. S. , Pascoal, D. O. , Nacif‐Pimenta, R. , Rodriguez, I. C. , Guerra, M. G. V. B. , Lacerda, M. V. G. , Secundino, N. F. C. , Monteiro, W. M. , & Pimenta, P. F. P. (2018). The role of the peritrophic matrix and red blood cell concentration in *plasmodium vivax* infection of *Anopheles aquasalis* . Parasites & Vectors, 11(1), 148. 10.1186/s13071-018-2752-5 29510729 PMC5840820

[mec17240-bib-0009] Beerntsen, B. , James, A. , & Christensen, B. (2000). Genetics of mosquito vector competence. Microbiology and Molecular Biology Reviews, 64(1), 115–137. 10704476 10.1128/mmbr.64.1.115-137.2000 PMC98988

[mec17240-bib-0010] Bensch, S. , Hellgren, O. , & Pérez‐Tris, J. (2009). MalAvi: A public database of malaria parasites and related haemosporidians in avian hosts based on mitochondrial cytochrome b lineages. Molecular Ecology Resources, 9(5), 1353–1358. 21564906 10.1111/j.1755-0998.2009.02692.x

[mec17240-bib-0011] Bolger, A. , Lohse, M. , & Usadel, B. (2014). Trimmomatic: a flexible trimmer for Illumina sequence data. Bioinformatics, 30(15), 2114–2120. 24695404 10.1093/bioinformatics/btu170 PMC4103590

[mec17240-bib-0012] Bonizzoni, M. , Dunn, W. , Campbell, C. , Olson, K. , Marinotti, O. , & James, A. A. (2012). Strain variation in the transcriptome of the dengue fever vector, *Aedes aegypti* . G3 (Bethesda, Md), 2(1), 103–114. 10.1534/g3.111.001107 22384387 PMC3276191

[mec17240-bib-0013] Bonizzoni, M. , Gasperi, G. , Chen, X. , & James, A. (2013). The invasive mosquito species *Aedes albopictus*: Current knowledge and future perspectives. Trends in Parasitology, 29(9), 460–468. 23916878 10.1016/j.pt.2013.07.003 PMC3777778

[mec17240-bib-0014] Bonnot, T. , Gillard, M. , & Nagel, D. (2019). A simple protocol for informative visualization of enriched gene ontology terms. Bio‐Protocol, 9(22), e3429. 10.21769/bioprotoc.3429

[mec17240-bib-0015] Borges‐Veloso, A. , Saboia‐Vahia, L. , Cuervo, P. , Pires, R. , Britto, C. , Fernandes, N. , d'Avila‐Levy, C. M. , & De Jesus, J. B. (2012). Proteolytic profiling and comparative analyses of active trypsin‐like serine peptidases in preimaginal stages of *Culex quinquefasciatus* . Parasites & Vectors, 5(1), 1–11. 10.1186/1756-3305-5-123 22892097 PMC3453504

[mec17240-bib-0016] Borovsky, D. , & Schlein, Y. (1987). Trypsin and chymotrypsin‐Iike enzymes of the sandfly *Phlebotomus papatasi* infected with *Leishmania* and their possible role in vector competence. Medical and Veterinary Entomology, 1(3), 235–242. 2979536 10.1111/j.1365-2915.1987.tb00349.x

[mec17240-bib-0017] Borovsky, D. , & Schlein, Y. (1988). Quantitative determination of trypsinlike and chymotrypsinlike enzymes in insects. Archives of Insect Biochemistry and Physiology, 8(4), 249–260.

[mec17240-bib-0018] Cirimotich, C. , Dong, Y. , Garver, L. , Sim, S. , & Dimopoulos, G. (2010). Mosquito immune defenses against *plasmodium* infection. Developmental and Comparative Immunology, 34(4), 387–395. 20026176 10.1016/j.dci.2009.12.005 PMC3462653

[mec17240-bib-0019] Clayton, A. M. , Dong, Y. , & Dimopoulos, G. (2014). The anopheles innate immune system in the defense against malaria infection. Journal of Innate Immunity, 6, 169–181. 23988482 10.1159/000353602 PMC3939431

[mec17240-bib-0020] Dana, A. N. , Hong, Y. , Kern, M. K. , Hillenmeyer, M. E. , Harker, B. , Lobo, N. F. , Hogan, J. R. , Romans, P. , & Collins, F. H. (2005). Gene expression patterns associated with blood‐feeding in the malaria mosquito *Anopheles gambiae* . BMC Genetics, 6(1), 5. 10.1186/1471-2164-6-5 15651988 PMC546002

[mec17240-bib-0021] De Gregorio, E. (2002). The toll and Imd pathways are the major regulators of the immune response in *Drosophila* . The EMBO Journal, 21(11), 2568–2579. 12032070 10.1093/emboj/21.11.2568 PMC126042

[mec17240-bib-0022] de Swart, M. M. , Balvers, C. , Verhulst, N. O. , & Koenraadt, C. J. M. (2023). Effects of host blood on mosquito reproduction. Trends in Parasitology, 39(7), 575–587. 37230833 10.1016/j.pt.2023.04.003

[mec17240-bib-0023] Detinova, T. S. , Bertram, D. S. , & World Health Organization . (1962). Age‐grouping methods in diptera of medical importance, with special reference to some vectors of malaria/T. S. Detinova; [with] an Annex on the ovary and ovarioles of mosquitos (with glossary) by D. S. Bertram. World Health Organization. 13885800

[mec17240-bib-0024] Dobin, A. , Davis, C. , Schlesinger, F. , Drenkow, J. , Zaleski, C. , Jha, S. , Batut, P. , Chaisson, M. , & Gingeras, T. R. (2012). STAR: ultrafast universal RNA‐seq aligner. Bioinformatics, 29(1), 15–21. 23104886 10.1093/bioinformatics/bts635 PMC3530905

[mec17240-bib-0025] Etebari, K. , Hegde, S. , Saldaña, M. , Widen, S. , Wood, T. , Asgari, S. , & Hughes, G. L. (2017). Global transcriptome analysis of *Aedes aegypti* mosquitoes in response to zika virus infection. MSphere, 2(6), e00456‐17. 10.1128/mSphere.00456-17 29202041 PMC5700376

[mec17240-bib-0026] Ewels, P. , Magnusson, M. , Lundin, S. , & Käller, M. (2016). MultiQC: Summarize analysis results for multiple tools and samples in a single report. Bioinformatics, 32(19), 3047–3048. 27312411 10.1093/bioinformatics/btw354 PMC5039924

[mec17240-bib-0027] Ferreira, F. C. , Videvall, E. , Seidl, C. M. , Wagner, N. E. , Kilpatrick, A. M. , Fleischer, R. C. , & Fonseca, D. M. (2022). Transcriptional response of individual Hawaiian *Culex quinquefasciatus* mosquitoes to the avian malaria *parasite Plasmodium relictum* . Malaria Journal, 21(1), 249. 10.1186/s12936-022-04271-x 36038897 PMC9422152

[mec17240-bib-0028] Foo, A. , Thompson, P. , Chen, S.‐H. , Jadi, R. , Lupo, B. , DeRose, E. F. , Arora, S. , Placentra, V. C. , Premkumar, L. , Perera, L. , Pedersen, L. C. , Martin, N. , & Mueller, G. A. (2021). The mosquito protein AEG12 displays both cytolytic and antiviral properties via a common lipid transfer mechanism. Proceedings of the National Academy of Sciences of the United States of America, 118(11), e2019251118. 33688047 10.1073/pnas.2019251118 PMC7980415

[mec17240-bib-0029] Frolet, C. , Thoma, M. , Blandin, S. , Hoffmann, J. A. , & Levashina, E. A. (2006). Boosting NF‐kappaB‐dependent basal immunity of *Anopheles gambiae* aborts development of *Plasmodium berghei* . Immunity, 25(4), 677–685. 10.1016/j.immuni.2006.08.019 17045818

[mec17240-bib-0030] García‐Longoria, L. , Ahrén, D. , Berthomieu, A. , Kalbskopf, V. , Rivero, A. , & Hellgren, O. (2022). Immune gene expression in the mosquito vector *Culex quinquefasciatus* during an avian malaria infection. Molecular Ecology, 32, 904–919. 10.1111/mec.16799 36448733 PMC10108303

[mec17240-bib-0031] García‐Longoria, L. , Palinauskas, V. , Ilgūnas, M. , Valkiūnas, G. , & Hellgren, O. (2020). Differential gene expression of *Plasmodium homocircumflexum* (lineage pCOLL4) across two experimentally infected passerine bird species. Genomics, 112(4), 2857–2865. 32234432 10.1016/j.ygeno.2020.03.025

[mec17240-bib-0032] Gemmell, N. , & Akiyama, S. (1996). An efficient method for the extraction of DNA from vertebrate tissues. Trends in Genetics, 12(9), 338–339. 8855658 10.1016/s0168-9525(96)80005-9

[mec17240-bib-0033] Giraldo‐Calderón, G. , Emrich, S. , MacCallum, R. , Maslen, G. , Dialynas, E. , Topalis, P. , Ho, N. , Gesing, S. , VectorBase Consortium , Madey, G. , Collins, F. H. , & Lawson, D. (2015). VectorBase: An updated bioinformatics resource for invertebrate vectors and other organisms related with human diseases. Nucleic Acids Research, 43(D1), D707–D713. 25510499 10.1093/nar/gku1117 PMC4383932

[mec17240-bib-0034] Gunay, F. , Picard, M. , & Robert, V. (2018). MosKeyTool, an interactive identification key for mosquitoes of Euro‐Mediterranean. Version 2.1. 2018. http://www.medilabsecure.com/moskeytool

[mec17240-bib-0035] Gutiérrez‐López, R. , Martínez‐de la Puente, J. , Gangoso, L. , Soriguer, R. , & Figuerola, J. (2020). *Plasmodium* transmission differs between mosquito species and parasite lineages. Parasitology, 147(4), 441–447. 31965951 10.1017/S0031182020000062 PMC10317650

[mec17240-bib-0036] Gwadz, R. , Kaslow, D. , Lee, J. , Maloy, W. , Zasloff, M. , & Miller, L. H. (1989). Effects of magainins and cecropins on the sporogonic development of malaria parasites in mosquitoes. Infection and Immunity, 57(9), 2628–2633. 2759705 10.1128/iai.57.9.2628-2633.1989 PMC313504

[mec17240-bib-0037] Han, Y. S. (2000). Molecular interactions between Anopheles stephensi midgut cells and *Plasmodium berghei*: The time bomb theory of ookinete invasion of mosquitoes. The EMBO Journal, 19(22), 6030–6040. 11080150 10.1093/emboj/19.22.6030 PMC305834

[mec17240-bib-0038] Hegedus, D. , Erlandson, M. , Gillott, C. , & Toprak, U. (2009). New insights into peritrophic matrix synthesis, architecture, and function. Annual Review of Entomology, 54(1), 285–302. 10.1146/annurev.ento.54.110807.090559 19067633

[mec17240-bib-0039] Hellgren, O. , Waldenström, J. , & Bensch, S. (2004). A new PCR assay for simultaneous studies of leucocytozoon, *Plasmodium*, and *Haemoproteus* from avian blood. The Journal of Parasitology, 90(4), 797–802. 15357072 10.1645/GE-184R1

[mec17240-bib-0040] Hernandez‐Caballero, I. , Hellgren, O. , & Garcia‐Longoria, L. (2023). Genomic advances in mosquito vector during avian malaria infection. Parasitology, 1–10. 10.1017/s0031182023000756 PMC10941221 37614176

[mec17240-bib-0041] Higgs, S. , & Beaty, B. J. (2005). Natural cycles of vector‐borne pathogens. Biology of disease vectors. In W. C. Marquardt , et al. (Eds.), Biology of disease vectors (2nd ed.). Elsevier Academic Press.

[mec17240-bib-0042] Hillyer, J. (2016). Insect immunology and hematopoiesis. Developmental and Comparative Immunology, 58, 102–118. 26695127 10.1016/j.dci.2015.12.006 PMC4775421

[mec17240-bib-0094] Hillyer, J. F. , Barreau, C. , & Vernick, K. D. (2007). Efficiency of salivary gland invasion by malaria sporozoites is controlled by rapid sporozoite destruction in the mosquito haemocoel. International Journal for Parasitology, 37(6), 673–681. 10.1016/j.ijpara.2006.12.007 17275826 PMC1905829

[mec17240-bib-0043] Huber, W. , Carey, V. , Gentleman, R. , Anders, S. , Carlson, M. , Carvalho, B. S. , Bravo, H. C. , Davis, S. , Gatto, L. , Girke, T. , Gottardo, R. , Hahne, F. , Hansen, K. D. , Irizarry, R. A. , Lawrence, M. , Love, M. I. , MacDonald, J. , Obenchain, V. , Oleś, A. K. , … Morgan, M. (2015). Orchestrating high‐throughput genomic analysis with Bioconductor. Nature Methods, 12(2), 115–121. 25633503 10.1038/nmeth.3252 PMC4509590

[mec17240-bib-0044] Jaynes, J. , Burton, C. , Barr, S. , Jeffers, G. , Julian, G. , White, K. L. , Enright, F. M. , Klei, T. R. , & Laine, R. A. (1988). In vitro cytocidal effect of novel lytic peptides on *plasmodium* falciparum and *Trypanosoma cruzi* . The FASEB Journal, 2(13), 2878–2883. 3049204 10.1096/fasebj.2.13.3049204

[mec17240-bib-0045] Jiang, H. , Vilcinskas, A. , & Kanost, M. R. (2010). Immunity in lepidopteran insects. Advances in Experimental Medicine and Biology, 708, 181–204. 10.1007/978-1-4419-8059-5_10 21528699 PMC9284565

[mec17240-bib-0046] Kazlauskienė, R. , Bernotienė, R. , Palinauskas, V. , Iezhova, T. A. , & Valkiūnas, G. (2013). *Plasmodium relictum* (lineages pSGS1 and pGRW11): Complete synchronous sporogony in mosquitoes *Culex pipiens* . Experimental Parasitology, 133(4), 454–461. 10.1016/j.exppara.2013.01.008 23337824

[mec17240-bib-0047] Kearse, M. , Moir, R. , Wilson, A. , Stones‐Havas, S. , Cheung, M. , Sturrock, S. , Buxton, S. , Cooper, A. , Markowitz, S. , Duran, C. , Thierer, T. , Ashton, B. , Meintjes, P. , & Drummond, A. (2012). Geneious basic: An integrated and extendable desktop software platform for the organization and analysis of sequence data. Bioinformatics, 28(12), 1647–1649. 22543367 10.1093/bioinformatics/bts199 PMC3371832

[mec17240-bib-0048] Kendziorski, C. , Irizarry, R. A. , Chen, K.‐S. , Haag, J. D. , & Gould, M. N. (2005). On the utility of pooling biological samples in microarray experiments. Proceedings of the National Academy of Sciences of the United States of America, 102(12), 4252–4257. 15755808 10.1073/pnas.0500607102 PMC552978

[mec17240-bib-0049] Kim, W. , Koo, H. , Richman, A. , Seeley, D. , Vizioli, J. , Klocko, A. D. , & O'Brochta, D. A. (2004). Ectopic expression of a cecropin transgene in the human malaria vector mosquito *Anopheles gambiae* (Diptera: Culicidae): Effects on susceptibility to *Plasmodium* . Journal of Medical Entomology, 41(3), 447–455. 15185949 10.1603/0022-2585-41.3.447

[mec17240-bib-0050] Korshunov, S. S. , Skulachev, V. P. , & Starkov, A. A. (1997). High protonic potential actuates a mechanism of production of reactive oxygen species in mitochondria. FEBS Letters, 416, 15–18. 9369223 10.1016/s0014-5793(97)01159-9

[mec17240-bib-0051] Kowaltowski, A. , de Souza‐Pinto, N. , & Castilho, V. A. (2009). Mitochondria and reactive oxygen species. Free Radical Biology & Medicine, 47(4), 333–343. 19427899 10.1016/j.freeradbiomed.2009.05.004

[mec17240-bib-0052] Kumar, A. , Srivastava, P. , Sirisena, P. , Dubey, S. , Kumar, R. , Shrinet, J. , & Sunil, S. (2018). Mosquito Innate Immunity. Insects, 9(3), 95. 30096752 10.3390/insects9030095 PMC6165528

[mec17240-bib-0053] Kumar, M. , Mohanty, A. , Sreenivasamurthy, S. , Dey, G. , Advani, J. , Pinto, S. M. , Kumar, A. , & Prasad, T. S. K. (2017). Response to blood meal in the fat body of *Anopheles stephensi* using quantitative proteomics: Toward new vector control strategies against malaria. OMICS: A Journal of Integrative Biology, 21(9), 520–530. 28873011 10.1089/omi.2017.0092

[mec17240-bib-0054] Kumar, S. , Christophides, G. K. , Cantera, R. , Charles, B. , Han, Y. S. , Meister, S. , Dimopoulos, G. , Kafatos, F. C. , & Barillas‐Mury, C. (2003). The role of reactive oxygen species on *Plasmodium* melanotic encapsulation in Anopheles gambiae. Proceedings of the National Academy of Sciences of the United States of America, 100(24), 14139–14144. 14623973 10.1073/pnas.2036262100 PMC283559

[mec17240-bib-0055] Lehane, M. (1997). Peritrophic matrix structure and function. Annual Review of Entomology, 42(1), 525–550. 10.1146/annurev.ento.42.1.525 15012322

[mec17240-bib-0056] Lehane, M. J. (2010). The biology of blood‐sucking in insects (2nd ed.). Cambridge University Press.

[mec17240-bib-0057] Li, B. , & Dewey, C. (2011). RSEM: Accurate transcript quantification from RNA‐seq data with or without a reference genome. BMC Bioinformatics, 12(1), 323. 10.1186/1471-2105-12-323 21816040 PMC3163565

[mec17240-bib-0058] Love, M. , Huber, W. , & Anders, S. (2014). Moderated estimation of fold change and dispersion for RNA‐seq data with DESeq2. Genome Biology, 15(12), 550. 10.1186/s13059-014-0550-8 25516281 PMC4302049

[mec17240-bib-0059] Luckhart, S. , Crampton, A. L. , Zamora, R. , Lieber, M. J. , dos Santos, P. , Peterson, T. M. , Emmith, N. , Lim, J. , Wink, D. A. , & Vodovotz, Y. (2003). Mammalian transforming growth factor β1 activated after ingestion by *Anopheles stephensi* modulates mosquito immunity. Infection and Immunity, 71(6), 3000–3009. 12761076 10.1128/IAI.71.6.3000-3009.2003 PMC155698

[mec17240-bib-0060] Memarpoor‐Yazdi, M. , Zare‐Zardini, H. , & Asoodeh, A. (2013). A novel antimicrobial peptide derived from the insect *Paederus dermatitis* . International Journal of Peptide Research and Therapeutics, 19(2), 99–108.

[mec17240-bib-0061] Michel, K. , & Kafatos, F. (2005). Mosquito immunity against *Plasmodium* . Insect Biochemistry and Molecular Biology, 35(7), 677–689. 15894185 10.1016/j.ibmb.2005.02.009

[mec17240-bib-0062] Molina‐Cruz, A. , Gupta, L. , Richardson, J. , Bennett, K. , Black, W., 4th , & Barillas‐Mury, C. (2005). Effect of mosquito midgut trypsin activity on dengue‐2 virus infection and dissemination in *Aedes aegypti* . The American Journal of Tropical Medicine and Hygiene, 72(5), 631–637. 15891140

[mec17240-bib-0063] Molina‐Cruz, A. , DeJong, R. J. , Charles, B. , Gupta, L. , Kumar, S. , Jaramillo‐Gutierrez, G. , & Barillas‐Mury, C. (2008). Reactive oxygen species modulate Anopheles gambiae immunity against bacteria and *Plasmodium* . The Journal of Biological Chemistry, 283, 3217–3223. 18065421 10.1074/jbc.M705873200

[mec17240-bib-0064] Morlais, I. , Mori, A. , Schneider, J. , & Severson, D. (2003). A targeted approach to the identification of candidate genes determining susceptibility to *Plasmodium gallinaceum* in *Aedes aegypti* . Molecular Genetics and Genomics, 269(6), 753–764. 14513362 10.1007/s00438-003-0882-7

[mec17240-bib-0065] Muller, H. , Catteruccia, F. , Vizioli, J. , Dellatorre, A. , & Crisanti, A. (1995). Constitutive and blood meal‐induced trypsin genes in *Anopheles gambiae* . Experimental Parasitology, 81(3), 371–385. 7498434 10.1006/expr.1995.1128

[mec17240-bib-0066] Nag, D. K. , Dieme, C. , Lapierre, P. , Lasek‐Nesselquist, E. , & Kramer, L. D. (2021). RNA‐seq analysis of blood meal induced gene‐expression changes in *Aedes aegypti* ovaries. BMC Genomics, 22(1), 396. 10.1186/s12864-021-07551-z 34044772 PMC8161926

[mec17240-bib-0067] Paxton, K. L. , Cassin‐Sackett, L. , Atkinson, C. T. , Videvall, E. , Campana, M. G. , & Fleischer, R. C. (2023). Gene expression reveals immune response strategies of naïve Hawaiian honeycreepers experimentally infected with introduced avian malaria. The Journal of Heredity, 114(4), 326–340. 36869776 10.1093/jhered/esad017

[mec17240-bib-0068] R Core Team . (2021). R: A language and environment for statistical computing. R Foundation for Statistical Computing. https://www.R‐project.org/

[mec17240-bib-0069] Sanders, H. R. , Evans, A. M. , Ross, L. S. , & Gill, S. S. (2003). Blood meal induces global changes in midgut gene expression in the disease vector, *Aedes aegypti* . Insect Biochemistry and Molecular Biology, 33(11), 1105–1122. 14563362 10.1016/s0965-1748(03)00124-3

[mec17240-bib-0070] Serrano‐Pinto, V. , Acosta‐Pérez, M. , Luviano‐Bazán, D. , Hurtado‐Sil, G. , Batista, C. V. F. , Martínez‐Barnetche, J. , & Lánz‐Mendoza, H. (2010). Differential expression of proteins in the midgut of *Anopheles albimanus* infected with *Plasmodium berghei* . Insect Biochemistry and Molecular Biology, 40(10), 752–758. 20692341 10.1016/j.ibmb.2010.07.011

[mec17240-bib-0071] Shahabuddin, M. , Lemos, F. , Kaslow, D. , & Jacobs‐Lorena, M. (1996). Antibody mediated inhibition of *Aedes aegypti* midgut trypsins blocks sporogonic development of *Plasmodium gallinaceum* . Infection and Immunity, 64(3), 739–743. 8641775 10.1128/iai.64.3.739-743.1996 PMC173831

[mec17240-bib-0072] Shao, L. , Devenport, M. , Fujioka, H. , Ghosh, A. , & Jacobs‐Lorena, M. (2005). Identification and characterization of a novel peritrophic matrix protein, Ae‐Aper50, and the microvillar membrane protein, AEG12, from the mosquito, *Aedes aegypti* . Insect Biochemistry and Molecular Biology, 35(9), 947–959. 15978997 10.1016/j.ibmb.2005.03.012

[mec17240-bib-0073] Shen, W. , He, P. , Xiao, C. , & Chen, X. (2018). From antimicrobial peptides to antimicrobial poly(α‐amino acid)s. Advanced Healthcare Materials, 7(20), 1800354. 10.1002/adhm.201800354 29923332

[mec17240-bib-0074] Shia, A. , Glittenberg, M. , Thompson, G. , Weber, A. , Reichhart, J. , & Ligoxygakis, P. (2009). Toll‐dependent antimicrobial responses in *drosophila* larval fat body require Spätzle secreted by haemocytes. Journal of Cell Science, 122(24), 4505–4515. 10.1242/jcs.049155 19934223 PMC2787462

[mec17240-bib-0075] Shin, S. , Bian, G. , & Raikhel, A. (2006). A toll receptor and a cytokine, Toll5A and Spz1C, are involved in toll antifungal immune signaling in the mosquito *Aedes aegypti* . The Journal of Biological Chemistry, 281(51), 39388–39395. 17068331 10.1074/jbc.M608912200

[mec17240-bib-0076] Sinden, R. (1983). Sexual development of malarial parasites. Advances in Parasitology, 22, 153–216. 6141715 10.1016/s0065-308x(08)60462-5

[mec17240-bib-0077] Sinden, R. (2002). Molecular interactions between *Plasmodium* and its insect vectors. Cellular Microbiology, 4(11), 713–724. 12427094 10.1046/j.1462-5822.2002.00229.x

[mec17240-bib-0078] Therneau, T. , Hart, S. , & Kocher, J. (2023). Calculating samplesSize estimates for RNA seq studies. R package version 1.40.0.

[mec17240-bib-0079] Todd, E. V. , Black, M. A. , & Gemmell, N. J. (2016). The power and promise of RNA‐seq in ecology and evolution. Molecular Ecology, 25(6), 1224–1241. 26756714 10.1111/mec.13526

[mec17240-bib-0080] Valkiūnas, G. (2005). Avian malaria parasites and other Haemosporidia. CRC Press.

[mec17240-bib-0081] Valkiūnas, G. , & Iezhova, T. A. (2018). Keys to the avian malaria parasites. Malaria Journal, 17(1), 212. 10.1186/s12936-018-2359-5 29843718 PMC5975542

[mec17240-bib-0082] Valkiūnas, G. , Žiegytė, R. , Palinauskas, V. , Bernotienė, R. , Bukauskaitė, D. , Ilgūnas, M. , Dimitrov, D. , & Iezhova, T. A. (2015). Complete sporogony of *Plasmodium relictum* (lineage pGRW4) in mosquitoes *Culex pipiens*, with implications on avian malaria epidemiology. Parasitology Research, 114(8), 3075–3085. 10.1007/s00436-015-4510-3 25958156

[mec17240-bib-0083] van Riper, C. , van Riper, S. , Goff, M. , & Laird, M. (1986). The epizootiology and ecological significance of malaria in Hawaiian land birds. Ecological Monographs, 56(4), 327–344.

[mec17240-bib-0084] Videvall, E. , Cornwallis, C. , Ahrén, D. , Palinauskas, V. , Valkiūnas, G. , & Hellgren, O. (2017). The transcriptome of the avian malaria parasite *plasmodium ashfordi* displays host‐specific gene expression. Molecular Ecology, 26(11), 2939–2958. 28267239 10.1111/mec.14085

[mec17240-bib-0085] Videvall, E. , Cornwallis, C. K. , Palinauskas, V. , Valkiūnas, G. , & Hellgren, O. (2015). The avian transcriptome response to malaria infection. Molecular Biology and Evolution, 32(5), 1255–1267. 10.1093/molbev/msv016 25636457 PMC4408411

[mec17240-bib-0086] Videvall, E. , Paxton, K. , Campana, M. , Cassin‐Sackett, L. , Atkinson, C. , & Fleischer, R. C. (2021). Transcriptome assembly and differential gene expression of the invasive avian malaria parasite *plasmodium relictum* in Hawaiʻi. Ecology and Evolution, 11(9), 4935–4944. 33976860 10.1002/ece3.7401 PMC8093664

[mec17240-bib-0087] Viljakainen, L. (2015). Evolutionary genetics of insect innate immunity. Briefings in Functional Genomics, 14(6), 407–412. 25750410 10.1093/bfgp/elv002 PMC4652032

[mec17240-bib-0088] Vizioli, J. , Bulet, P. , Charlet, M. , Lowenberger, C. , Blass, C. , Müller, H. M. , Dimopoulos, G. , Hoffmann, J. , Kafatos, F. C. , & Richman, A. (2000). Cloning and analysis of a cecropin gene from the malaria vector mosquito, *Anopheles gambiae* . Insect Molecular Biology, 9(1), 75–84. 10672074 10.1046/j.1365-2583.2000.00164.x

[mec17240-bib-0089] Vizioli, J. , Catteruccia, F. , Della Torre, A. , Reckmann, I. , & Müller, H. (2001). Blood digestion in the malaria mosquito *Anopheles gambiae* . European Journal of Biochemistry, 268(14), 4027–4035. 11453997 10.1046/j.1432-1327.2001.02315.x

[mec17240-bib-0090] Vlachou, D. , Schlegelmilch, T. , Christophides, G. , & Kafatos, F. (2005). Functional genomic analysis of midgut epithelial responses in *Anopheles* during *Plasmodium* invasion. Current Biology, 15(13), 1185–1195. 16005290 10.1016/j.cub.2005.06.044

[mec17240-bib-0091] WHO . (2021). World malaria report 2021: regional data and trends. In World Health: Vol. WHO/UCN/GMP/2021.09 (Issue December). https://www.who.int/publications/m/item/WHO‐UCN‐GMP‐2021.09

[mec17240-bib-0092] Wickham, H. (2016). ggplot2: Elegant graphics for data analysis. Springer‐Verlag.

[mec17240-bib-0093] Zou, Z. , Souza‐Neto, J. , Xi, Z. , Kokoza, V. , Shin, S. , Dimopoulos, G. , & Raikhel, A. (2011). Transcriptome analysis of *Aedes aegypti* transgenic mosquitoes with altered immunity. PLoS Pathogens, 7(11), e1002394. 10.1371/journal.ppat.1002394 22114564 PMC3219725

