# Supplementary material for: Two avian Plasmodium species trigger different transcriptional responses on their vector Culex pipiens
Source: Mol Ecol. 2023 Dec 18;34(15):e17240. doi: 10.1111/mec.17240 (PMC12288828; doi:10.1111/mec.17240)
Supplement: Supplementary file 1 — Supplementary Material S1. [file MEC-34-e17240-s001.docx]

**Two avian *Plasmodium* species trigger different transcriptional responses on their vector *Culex pipiens***

Garrigós M^1,2*^, Ylla G^3^, Martínez-de la Puente J^2,4^, Figuerola J^1,4^, Ruiz-López MJ^1,4^.

## **Contents**

## S1. 1. Methodological details of data processing

## S1. 2. Differentially Expressed Genes Analysis between time points

## S1. 3 Tables and Figures

## **S1. 1. Data processing methodological details**

We used Anaconda (ver. 3-5.0.1) to install all software used in data processing. We used FastQC (ver. 0.11.9) and MultiQC (ver. 0.9.1a0) to check the quality of the raw and filtered reads. We filtered low quality and under 36 bp reads using Trimmomatic (ver. 0.36) with the parameters *PE -phred33* *LEADING:3 TRAILING:3 MINLEN:50*. We used STAR (ver. 2.7.10a) to map the short reads to the reference genome of *Cx. quinquefasciatus* and RSEM (ver. 1.2.28) to quantify gene abundances. To do so, we first create the genome index using the script *rsem-prepare-reference* with the parameters *--gtf* *and --star*. We then mapped the reads and quantified the gene expression in a single step using the script *rsem-calculate-expression* with the parameters *-p 20 --paired-end --star --star-gzipped-read-file --star-output-genome-bam*. Finally, we merged the individual gene counts for each sample into a single table of counts using the script *rsem-generate-data-matrix*.

## **S1. 2. Differentially Expressed Genes between time points**

We found a total of 6,125 differentially expressed genes between 24 hours post-infection (hpi) and 10 days post-infection (dpi) and 6,422 between 24 hpi and 21 dpi, of which 5,436 were coincident. By contrast, only 28 genes were differentially expressed between 10 dpi and 21 dpi (Figure S1).

Among the most up-regulated genes at 24 hpi compared to 10 dpi and 21 dpi we found three cathepsin B precursors (CPIJ015762, CPIJ015761, CPIJ000577), five protein G12 precursors (CPIJ012846, CPIJ012844, CPIJ005176, CPIJ012848, CPIJ012845) four vitellogenin-A1 precursors (CPIJ010191, CPIJ010190, CPIJ001358, CPIJ001357), and a vitelline membrane protein homolog (CPIJ018485) genes, and several serine- proteases and salivary enzymes including trypsin (CPIJ004660, CPIJ018529, CPIJ006019, CPIJ006018, CPIJ018339, CPIJ005132) and chymotrypsin (CPIJ015161, CPIJ014254, CPIJ013043) precursors, salivary apyrase (CPIJ018314), and the Salivary C-type lectin (CPIJ013150) genes, among others. On the other hand, down-regulated genes included 19 spliceosomal RNA (e.g. CPIJ039777, CPIJ039803, CPIJ039786, CPIJ039603, CPIJ039754) and more than 30 histone genes (e.g. CPIJ012416, CPIJ039919, CPIJ012428, CPIJ012436, CPIJ012440, CPIJ012444), among others.

At 10 dpi compared to 21 dpi up- regulated genes products included two ribosomal proteins (CPIJ013899, CPIJ018688), and down-regulated genes were mostly histones genes (e.g. CPIJ012428, CPIJ012432, CPIJ012436, CPIJ012440, CPIJ012444).

Enrichment analyses revealed very similar enriched GO terms for 24 hpi vs 10 dpi and 24 hpi vs 21 dpi. In both contrasts, at 24 hpi up-regulated genes were mainly involved in biological processes such as lipid, carbohydrate, protein and nucleotide metabolic processes, and molecular functions like peptidase and hydrolase activities. Down-regulated genes were related to cytoskeleton organization, chromatin assembly and RNA splicing biological processes, among others and nucleotide binding molecular functions.

## **S1. 3. Tables and Figures**

**Table S1**: General information of samples and their reads, including infection status, time post-infection, number of raw reads, and number and percentage of clean reads and reads mapped to Culex quinquefasciatus. Shaded samples were removed for downstream analyses because they presented barcoding errors due to lab processing.

| **Sample** | **Infection**  **status** | **Time** | **Raw reads** | **Clean reads (%)** | **Reads mapped to**  ***Culex quinquefasciatus* (%)** |
| --- | --- | --- | --- | --- | --- |
|  |  |  |  |  |  |
| T1-1-51 | Control | 24h | 33177082 | 31008365 (93.46) | 26038949  (83.98) |
| T1-2-51 | Control | 24h | 35149742 | 34069123 (96.93) | 28672552  (84.16) |
| T1-3-51 | Control | 24h | 32243129 | 30936498 (95.95) | 25993214  (84.02) |
| T1-4-51 | Control | 24h | 22466452 | 21294753 (94.78) | 16645600  (78.17) |
| T1-1-67 | *P. cathemerium* | 24h | 22498217 | 21936666 (97.50) | 18332032  (83.57) |
| T1-2-67 | *P. cathemerium* | 24h | 27397567 | 27128906 (99.02) | 22874928  (84.32) |
| T1-3-67 | *P. cathemerium* | 24h | 33324219 | 33162522 (99.51) | 27433799  (82.72) |
| T1-4-67 | *P. cathemerium* | 24h | 26626804 | 26462620 (99.38) | 21953450  (82.96) |
| T1-1-85 | *P. relictum* | 24h | 30674241 | 30435998 (99.22) | 24801694  (81.49) |
| T1-2-85 | *P. relictum* | 24h | 23278856 | 22883101 (98.30) | 19001869  (83.04) |
| T1-3-85 | *P. relictum* | 24h | 35771588 | 34263353 (95.78) | 28608913  (83.50) |
| T1-4-85 | *P. relictum* | 24h | 30472866 | 29351327 (96.32) | 24186857  (82.41) |
| T2-5-51 | Control | 10d | 28177843 | 25003834 (88.74) | 20239066  (80.95) |
| T2-16-51 | Control | 10d | 20967331 | 18808738 (89.70) | 15330448  (81.51) |
| T2-21-51 | Control | 10d | 25806437 | 22496937 (87.18) | 18230162  (81.04) |
| T2-15-51 | Control | 10d | 15406828 | 12411723 (80.56) | 9924380  (79.96) |
| T2-13-67 | *P. cathemerium* | 10d | 27298353 | 25826907 (94.61) | 19709378  (76.31) |
| T2-16-67 | *P. cathemerium* | 10d | 29882582 | 28545072 (95.52) | 21510841  (75.36) |
| T2-23-67 | *P. cathemerium* | 10d | 25577894 | 25107302 (98.16) | 19754366  (78.68) |
| T2-24-67 | *P. cathemerium* | 10d | 29176625 | 29037230 (99.52) | 22315395  (76.85) |
| T2-13-85 | *P. relictum* | 10d | 37005797 | 35991229 (97.26) | 29906455  (83.10) |
| T2-16-85 | *P. relictum* | 10d | 29891263 | 28936485 (96.81) | 24051388  (83.12) |
| T2-5-85 | *P. relictum* | 10d | 34277655 | 32255293 (94.10) | 25436232  (78.86) |
| T2-6-85 | *P. relictum* | 10d | 32903444 | 31841055 (96.77) | 26529969  (83.32) |
| T3-11-51 | Control | 21d | 32385022 | 32118665 (99.18) | 26043488  (81.09) |
| T3-17-51 | Control | 21d | 32770699 | 32039074 (97.77) | 26111299  (81.50) |
| T3-18-51 | Control | 21d | 29728686 | 29263504 (98.44) | 20838799  (71.21) |
| T3-21-51 | Control | 21d | 30766572 | 30159889 (98.03) | 24335913  80.69) |
| T3-17-67 | *P. cathemerium* | 21d | 21530168 | 21145476 (98.21) | 16532428  (78.19) |
| T3-18-67 | *P. cathemerium* | 21d | 37716163 | 37090714 (98.34) | 30680752  (82.72) |
| T3-22-67 | *P. cathemerium* | 21d | 28765518 | 28497563 (99.07) | 23298862  (81.76) |
| T3-9-67 | *P. cathemerium* | 21d | 39203774 | 35121618 (89.59) | 27087303  (77.12) |
| T3-10-85 | *P. relictum* | 21d | 45332626 | 44887231 (99.02) | 36489870  (81.30) |
| T3-11-85 | *P. relictum* | 21d | 33120712 | 32798179 (99.03) | 27092510  (81.90) |
| T3-14-85 | *P. relictum* | 21d | 41161560 | 40739559 (98.97) | 32717880  (80.31) |
| T3-9-85 | *P. relictum* | 21d | 28181782 | 28034675 (99.48) | 21149517  (80.18) |

**Table S2.** Data on mortality of mosquitoes from each infection status at each time-point and the overall mortality percentages.

| **Infection status** | **Total fed mosquitoes** | **Dead mosquitoes at 24 hpi** | **Dead mosquitoes at 10 dpi** | **Dead mosquitoes at 21 dpi** | **Global mortality (%)** |
| --- | --- | --- | --- | --- | --- |
| *P. relictum* | 120 | 5 | 1 | 5 | 9.17 |
| *P. cathemerium* | 71 | 2 | 2 | 0 | 5.63 |
| Control | 126 | 3 | 2 | 3 | 6.35 |

**Table S3**: Estimation of sample size required to detect genes with Log Fold Change (LFC) of 0.25, 0.5, 0.75, and 1 with a statistical power of 80% and 90% for the dataset analyzed in this work.

| **Log Fold Change** | **Sample size by statistical power** | |
| --- | --- | --- |
|  | **80%** | **90%** |
| 1 | 4.35 | 5.54 |
| 0.75 | 6.67 | 8.50 |
| 0.5 | 12.71 | 16.20 |
| 0.25 | 42.00 | 53.47 |


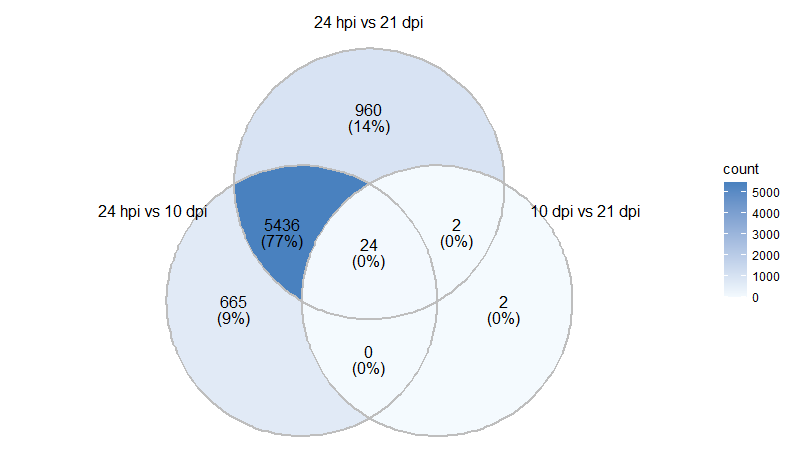


**Figure S1.** Venn diagram of the comparisons between time points regardless of infection status. The legend shows the number of genes in each intersection.


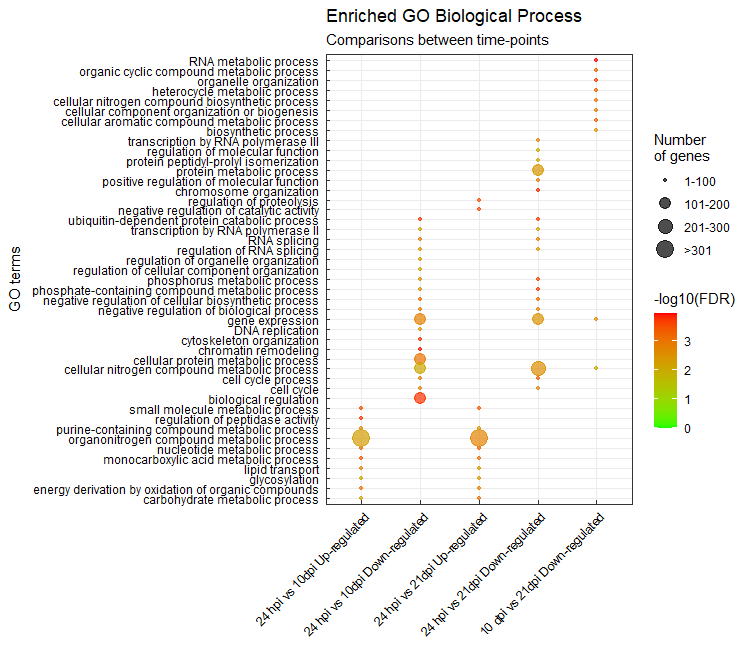


**Figure S2.** Dot plot of enriched GO biological processes for differentially expressed genes (up and down-regulated) at (1) 24 hours post-infection (hpi) vs 10 days post-infection (dpi), (2) 24 hpi vs 21 dpi, and (3) 10 dpi vs 21 dpi. The y-axis lists the GO terms. The legend shows both the number of significant genes (dot size) and the –log(p-value) (color gradient) for each GO term. Larger dots correspond to a higher number of significant genes, and the color gradient goes from green for the least significant terms to red for the most significant terms.

***
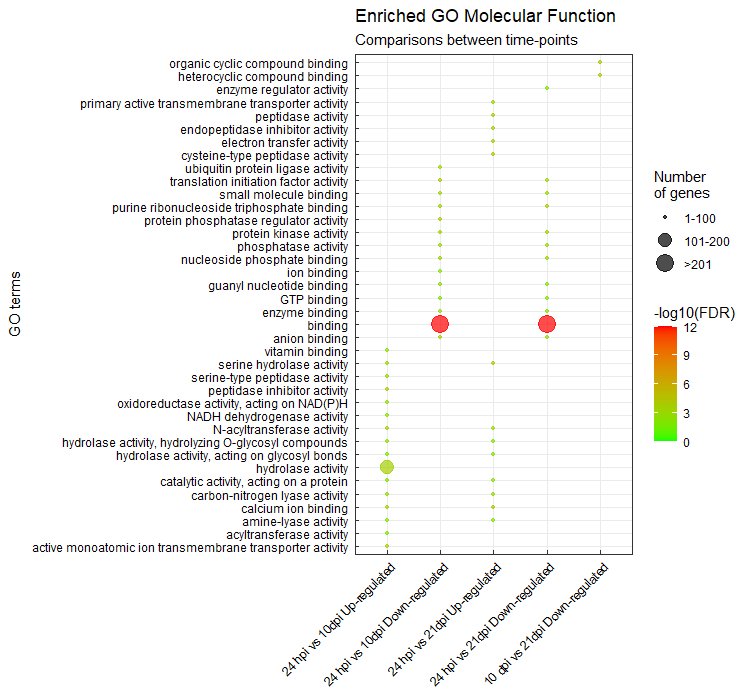
***

**Figure S3.** Dot plot of enriched GO molecular functions for differentially expressed genes (up and down-regulated) at (1) 24 hpi vs 10 dpi, (2) 24 hpi vs 21 dpi, and (3) 10 dpi vs 21 dpi.


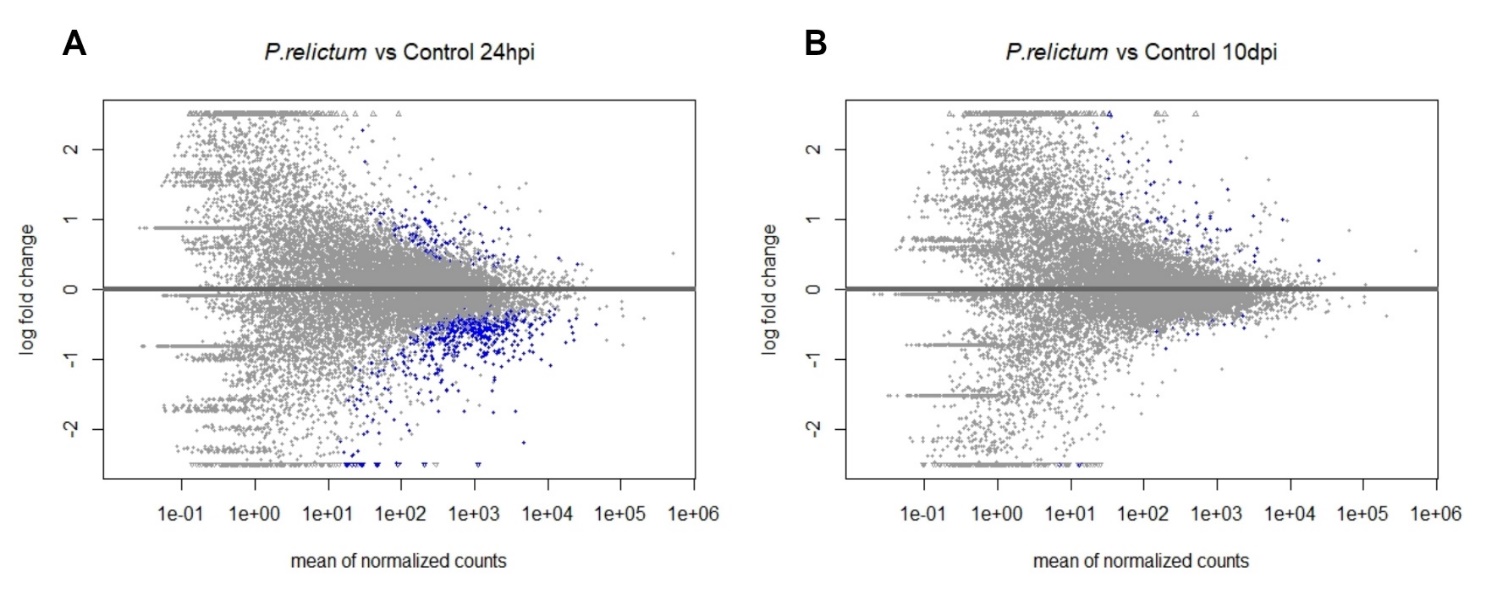
**Figure S4.** MA plot for gene expression of Plasmodium relictum–infected vs control mosquitoes at (A) 24 hpi and (B) 10 dpi. Triangles correspond to genes with a |LFC| > 1. Blue dots and triangles correspond to genes considered differentially expressed (adjusted p-value < 0.01). The y-axis shows the LFC (i.e. the intensity of the gene expression). The y-axis shows the average expression between the groups compared.

***
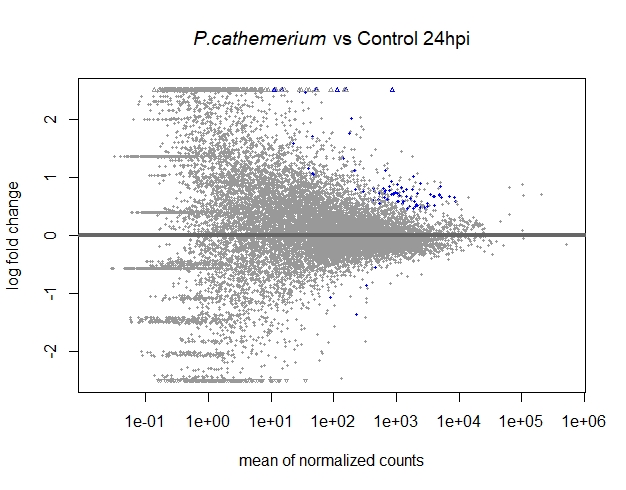
***

**Figure S5**. MA plot for gene expression of *Plasmodium cathemerium*- infected vs control mosquitoes at 24 hpi.

**
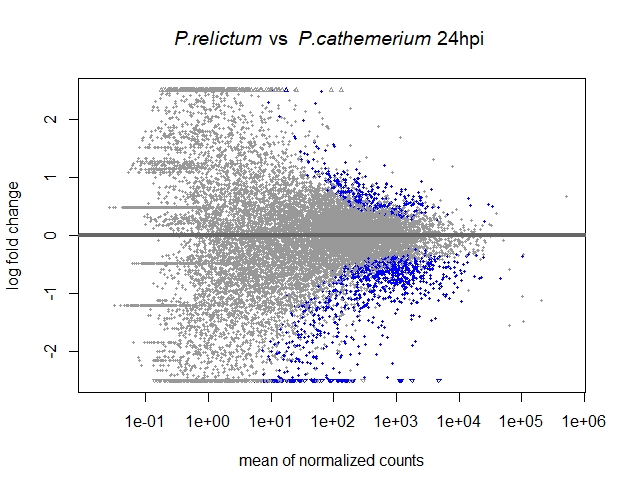
**

**Figure S6**. MA plot for gene expression of Plasmodium relictum vs Plasmodium cathemerium- infected mosquitoes at 24 hpi.
